# Insights into the *ANKRD11* variants and short-stature phenotype through literature review and ClinVar database search

**DOI:** 10.1186/s13023-024-03301-y

**Published:** 2024-08-12

**Authors:** Dongye He, Mei Zhang, Yanying Li, Fupeng Liu, Bo Ban

**Affiliations:** 1 Department of Endocrinology, Genetics and Metabolism, Affiliated Hospital of Jining Medical University, Jining, Shandong 272029 China; 2 Medical Research Center, Affiliated Hospital of Jining Medical University, Jining, China; 3 Chinese Research Center for Behavior Medicine in Growth and Development, Jining, China

**Keywords:** *ANKRD11* gene, KBG syndrome, Hotspot variants, Short stature, Growth hormone treatment, Growth plate development

## Abstract

Ankyrin repeat domain containing-protein 11 (ANKRD11), a transcriptional factor predominantly localized in the cell nucleus, plays a crucial role in the expression regulation of key genes by recruiting chromatin remodelers and interacting with specific transcriptional repressors or activators during numerous biological processes. Its pathogenic variants are strongly linked to the pathogenesis and progression of multisystem disorder known as KBG syndrome. With the widespread application of high-throughput DNA sequencing technologies in clinical medicine, numerous pathogenic variants in the *ANKRD11* gene have been reported. Patients with KBG syndrome usually exhibit a broad phenotypic spectrum with a variable degree of severity, even if having identical variants. In addition to distinctive dental, craniofacial and neurodevelopmental abnormalities, patients often present with skeletal anomalies, particularly postnatal short stature. The relationship between *ANKRD11* variants and short stature is not well-understood, with limited knowledge regarding its occurrence rate or underlying biological mechanism involved. This review aims to provide an updated analysis of the molecular spectrum associated with *ANKRD11* variants, investigate the prevalence of the short stature among patients harboring these variants, evaluate the efficacy of recombinant human growth hormone in treating children with short stature and *ANKRD11* variants, and explore the biological mechanisms underlying short stature from both scientific and clinical perspectives. Our investigation indicated that frameshift and nonsense were the most frequent types in 583 pathogenic or likely pathogenic variants identified in the *ANKRD11* gene. Among the 245 KBGS patients with height data, approximately 50% displayed short stature. Most patients showed a positive response to rhGH therapy, although the number of patients receiving treatment was limited. ANKRD11 deficiency potentially disrupts longitudinal bone growth by affecting the orderly differentiation of growth plate chondrocytes. Our review offers crucial insights into the association between *ANKRD11* variants and short stature and provides valuable guidance for precise clinical diagnosis and treatment of patients with KBG syndrome.

**Supplementary Information:**

The online version contains supplementary material available at 10.1186/s13023-024-03301-y.

## Background

The *ANKRD11* gene (OMIM#611192) is mapped to human chromosome 16q24.3 and encodes an ankyrin repeat domain-containing protein 11 that belongs to a member of the ankyrin repeats-containing cofactor family (ANCO). It is relatively conserved across species and ubiquitously expressed in multiple organs and tissues, particularly in the brain and ovary [1, 2]. The ANKRD11 protein, consisting of 2,663 amino acid residues, structurally includes the ankyrin domain (ANK), transcriptional activation domain (AD), transcriptional repression domains (RD1 and RD2), and multiple putative nuclear localization signals (NLSs) [3]. The N-terminal ANK domain follows the canonical helix-loop-helix-β-hairpin/loop configuration and is comprised of five consecutive ankyrin repeat motifs. Each motif contains a 33-residue sequence and facilitates protein-protein interaction to coordinate subsequent transcriptional regulatory processes [4–6]. The ANKRD11 protein binds to the conserved N-terminal Per-Arnt-Sim (PAS) region of p160 coactivator via its ANK domain, concurrently, recruits histone deacetylases (HDACs) through its RD1 or RD2 domain. When p160 coactivator binds to the hydrophobic cleft within the C-terminal ligand-binding domain (LBD) of nuclear receptors (NRs) through its LXXLL motifs, the assembly of p160/ANKRD11/HDACs complex suppresses NRs-mediated ligand-dependent transactivation [7]. The ANKRD11 protein also interacts with the N-terminal 84 amino acids of ADA3 (alteration/deficiency in activation 3), which is an essential part of the p300/CBP [cAMP-response-element binding protein-binding protein]-associated factor (P/CAF) complex. This complex connects coactivators to histone acetylation and basal transcription machinery, resulting in the recruitment of the P/CAF complex and the specific regulation of ADA3 coactivator in a transcription factor-dependent manner [8]. Moreover, the ANKRD11 protein is capable of amplifying p53 activity through the enhancement of P/CAF-mediated acetylation [6]. Overall, the ANKRD11 protein, through its various functional domains, collectively facilitates the formation of a molecular bridge between coactivators or corepressors and histone deacetylases (HDACs) or histone acetyltransferases (HATs), thereby precisely regulating the transcription of target genes.

Initially, *ANKRD11* has been recognized as a tumor suppressor gene in breast cancer due to its location within the chromosomal region 16q24.3, which is widely acknowledged for its frequent loss of heterozygosity (LOH) among patients suffering from breast cancer [9, 10]. Under normal physiological conditions, the estrogen receptor (ER)/amplified in breast cancer 1 (AIB1)/ANKRD11/HDACs or transcriptional enhanced associate domain (TEAD)/yes-associated protein (YAP)/AIB1/ANKRD11 complex functions to suppress the transcriptional activation of oncogenes in breast cancer [11, 12]. However, aberrant DNA methylation of three CpGs within a 19-base pair region of the *ANKRD11* promoter leads to its down-regulation, thereby disrupting the assembly of the complex and consequently promoting breast tumorigenesis [13]. ANKRD11 haploinsufficiency was later identified in KBG syndrome (KBGS) patient-focused clinical and molecular studies, confirming the dominant pathogenic mechanism responsible for this condition (OMIM#148050). KBGS was initially reported by Herrmann and colleagues in 1975 and characterized by macrodontia of the upper central incisors, distinctive craniofacial findings, postnatal short stature, skeletal anomalies and, neurodevelopmental disorders, sometimes with seizures and electroencephalogram (EEG) abnormalities [14–16]. Patients harboring *ANKRD11* pathogenic variants exhibit overlapping features between KBGS and Cornelia de Lange syndrome or Coffin-Siris-like syndrome, particularly neurological and skeletal anomalies [17, 18]. KBGS typically presents with a wide range of phenotypic manifestations, each varying in severity [19]. The biological function and cellular mechanism of *ANKRD11* variants associated with the KBGS features have garnered significant interest and attention within the academic community. Previous study has established the pivotal role of the *ANKRD11* gene in proliferation, neurogenesis and neuronal localization of cortical neural precursor cells by utilizing a Yoda mice model harboring a point mutation within the ANKRD11-HDAC interaction region, and the underlying mechanism was linked to alterations in the acetylation patterns of specific lysine residues (H3K9, H4K5, H4K8, H4K16) on the target genes regulated by ANKRD11 [20]. Further investigation has revealed that ANKRD11 regulates pyramidal neuron migration and dendritic differentiation of mouse cerebral cortex through the coordination of P/CAF to facilitate the acetylation of both p53 and Histone H3, which subsequently leads to the activation of brain-derived neurotrophic factor (BDNF)/tyrosine receptor kinase B (TrkB) signaling pathway [21]. Moreover, Roth and their colleagues developed a heterozygous neural crest-specific ANKRD11-mutant mice model, and revealed that multiple ossification centers in the middle facial bone of mice failed to expand or fuse properly, leading to a significant delay in bone maturation and a severe restriction in bone remodeling [22]. Recent research has uncovered that conditional knockout of the *ANKRD11* gene within murine embryonic neural crest leads to severe congenital cardiac malformations and the underlying mechanism was linked to a reduction in Sema3C expression levels, coupled with diminished mTOR and BMP signaling within the cardiac neural crest cells of the outflow tract [23]. Based on the accumulating evidence from ongoing research into gene functions, the relationship between *ANKRD11* pathogenic variants and the clinical features of KBGS is better understood than ever before. However, the role of *ANKRD11* variants in inducing short stature has not received sufficient attention, particularly regarding its frequency of occurrence and the underlying biological mechanisms of action.

## Materials and methods

We investigated publicly available online resources including published literature in Web of Science, PubMed, Google Scholar, and Wanfang database by searching keywords “KBGS”, “ANKRD11”, “Short stature” and “Intellectual disability” as well as genetic testing records in ClinVar database between July 2011 and March 2024. In this review, we included a total of 78 published papers that encompassed cohort studies, case series or single-case reports, and gathered 583 *ANKRD11* variants, which were classified as pathogenic or likely pathogenic according to the American College of Medical Genetics and Genomics (ACMG)-Association for Molecular Pathology (AMP) guideline (Supplemental material 1). Among these variants, 202 were reported in published papers and 381 were described in the ClinVar database. Certain large deletions or duplications of the *ANKRD11* gene were not considered in this analysis, as the complexity of their impact on the amino acid sequence of the encoded protein posed challenges for interpretation. We have also excluded patients with 16q24.3 microdeletions, 16q24.3 microduplications and dual molecular diagnosis involving *ANKRD11* and/or flanking genes, as the role of other genes in contributing to the height phenotype remains uncertain. Furthermore, hotspot variants within ANKRD11 were analyzed in 838 patients, comprising 457 derived from the literature and 381 derived from the ClinVar database (Supplemental material 2). *ANKRD11* allele frequency below 1% in the general poulation was obtained from gnomAD (http://gnomad-sg.org/). 245 patients were reported to have height data, of which 112 had a height SDS. The differences in height SDS among patients with short stature carrying various *ANKRD11* variants were further analyzed (Supplemental material 3). Data was described as mean ± SDS, and analyzed with one-way analysis of variance (ANOVA) followed by Tukey’s multiple comparisons test. A significant difference was considered when the *p*-value was less than 0.05.

### Molecular spectrum of *ANKRD11* variants

**Fig. 1:**
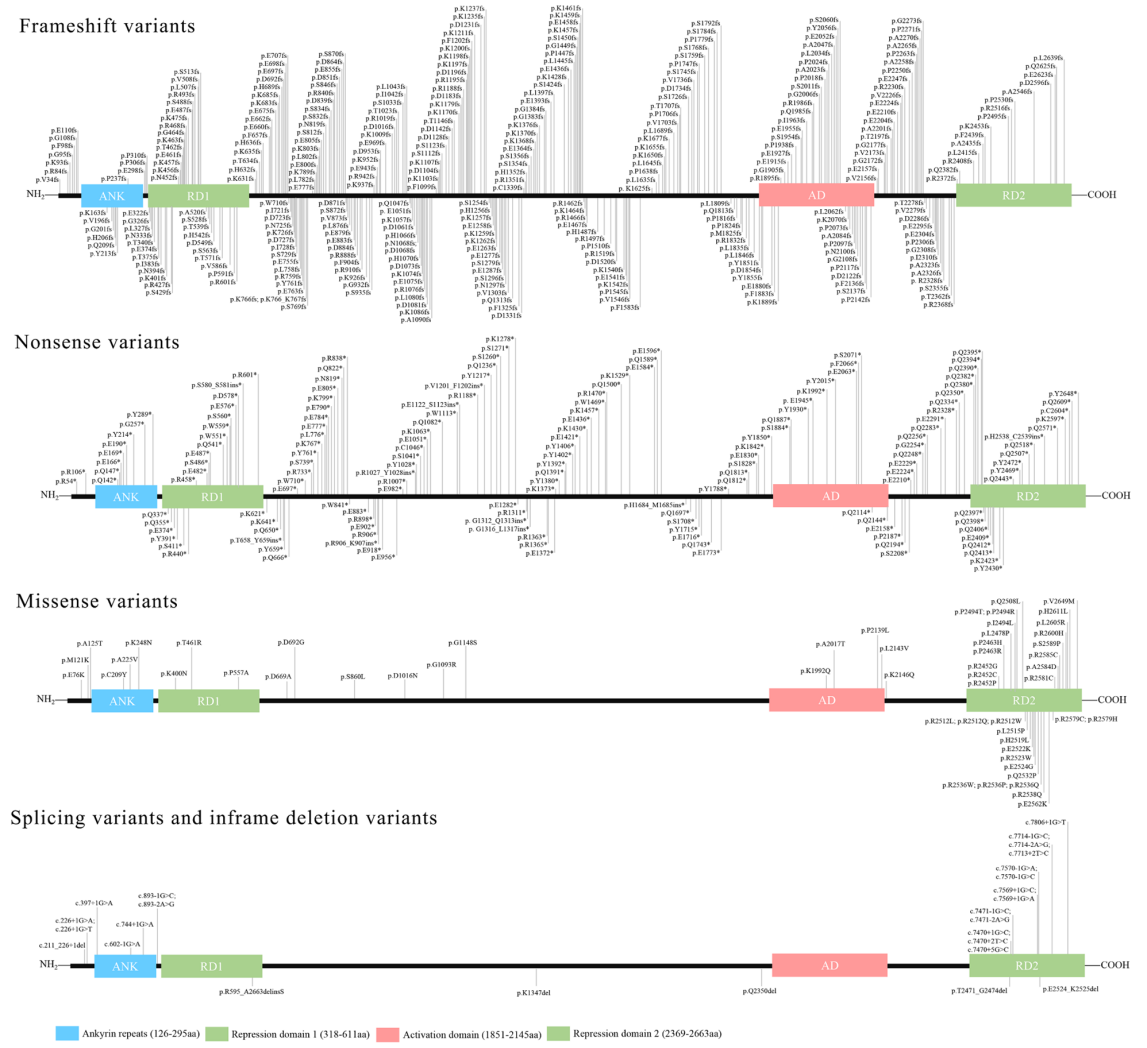
Molecular spectrum of *ANKRD11* variants. A total of 583 ANRKD11 (likely) pathogenic variants were collected through literature review and ClinVar database. *ANKRD11* variants were shown by frameshift, nonsense, missense, splice and inframe deletion, respectively. ANK: ankyrin repeat domain, RD1: repression domain 1, AD: activation domain, RD2: repression domain 2

Since *ANKRD11* was identified as the causal gene for KBGS in 2011, more than 340 KBGS patients have been reported worldwide [24]. Considering the variant data documented in the ClinVar database, it is projected that the number of patients with *ANKRD11* variants exceeds 800. Despite the global prevalence of KBGS worldwide remaining unknown, its prevalence is underestimated due to a limited understanding of the disease phenotype and molecular underpinning. Consequently, establishing the spectrum of genetic variation in the *ANKRD11* gene holds the promise of not only enhancing our understanding of disease’s pathogenesis but also enabling clinicians to render a precise molecular diagnosis for KBGS. A total of 583 *ANKRD11* variants encompassed nearly the entire sequence of amino acids [1, 2, 15, 17–19, 25–96] (Fig. 1). All identified *ANKRD11* variants were present in a heterozygous state, aligning with early embryonic lethality of Yoda mice observed in homozygotes, as demonstrated by Barbaric et al. [3]. This review encapsulates the up-to-date molecular landscape of *ANKRD11* variants, nevertheless, in light of the continual discovery of patients with newly identified *ANKRD11* variants, it needs to be supplemented and updated in time.

**Fig. 2:**
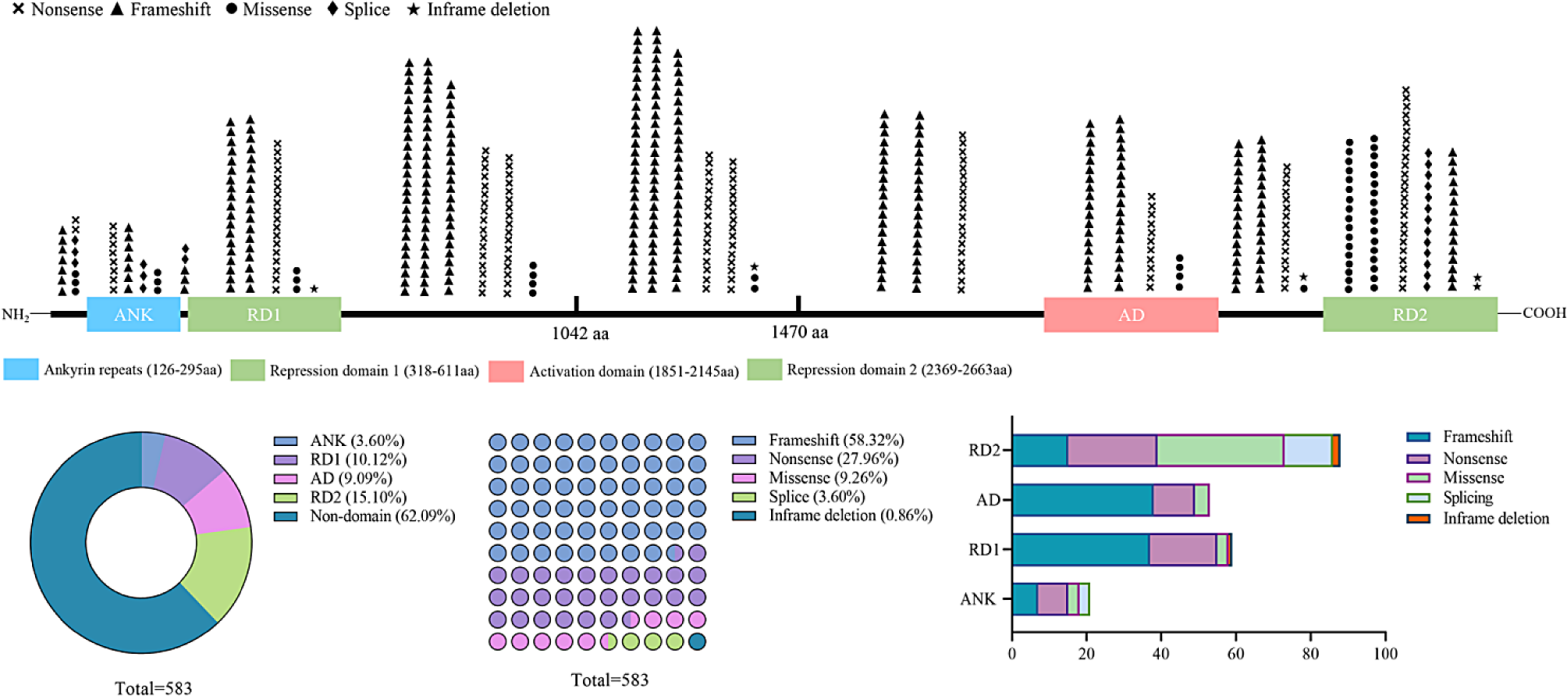
The percentage of different types of *ANKRD11* variants located in different functional domains. The pie chart indicates the percentage of variants within different domains. 10 X 10 dot plot represents the percentage of different variant types. The column shows the the proportion of five mutation types within different domains of ANKRD11. ANK: ankyrin repeat domain, RD1: repression domain 1, AD: activation domain, RD2: repression domain 2

All *ANKRD11* variants in the map were classified into five types: frameshift variants (340/583, 58.32%), nonsense variants (163/583, 27.96%), missense variants (54/583, 9.26%), splicing variants (21/583, 3.60%) and inframe deletion variants (5/583, 0.86%) (Fig. 2). Variants occurring in ANK, RD1, AD, RD2 and non-domain region accounted for 3.60%, 10.12%, 9.09%, 15.10% and 62.09% of the total variant pool, respectively (Fig. 2). Multiple putative NLSs within the interval between the RD1 and AD regions were categorized as part of the non-domain segment, primarily due to the absence of definitive and evidence-based localization data [3, 5, 15, 48]. Specific variants occurring within these NLSs may impair the nuclear targeting of the ANKRD11 protein. Notably, the most common variants were frameshift and nonsense variants, which give rise to prematurely truncated forms of the ANKRD11 protein. 62.96% (34/54) of *ANKRD11* missense variants were found to cluster within C-terminal RD2 region. The majority of these missense variants, particularly those impacting arginine residues, were reported to impair protein stability or transcriptional activity, however, they did not produce an obvious impact on the protein’s subcellular localization [61, 66]. Additionally, alternative splicing events predominantly affected the C-terminal RD2 (13/21) and N-terminal region (8/21). It is not surprising that those affecting 5’ and 3’ splice sites are commonly implicated as the underlying cause of hereditary disorders [97]. Nonetheless, how these hypothesized splicing variants impact the encoded protein requires an in-depth examination of splicing patterns by cDNA analysis, and frequently involves a Mini-gene assay. Other types of *ANKRD11* variants were relatively uncommon including p.Lys1347del, p.Thr2471_Gly2474del, p.Glu2524_Lys2525del, p.Q2350del, and p.R595_A2663delinsS. Interestingly, p.Lys1347del has been demonstrated to significantly disrupt the transcriptional activation of downstream *p21* gene but did not influence the levels of *ANKRD11* mRNA or protein [2, 15, 19, 61]. Theoretically, protein-truncating variants (PTVs) cause a more detrimental effect on protein function compared to the consequences of amino acid deletions (≥ 1) and single amino acid substitution [98, 99]. The impact of various types of genetic variants on the ANKRD11 protein function requires further investigation by a range of functional analyses.

### Hotspot variants of ANKRD11 protein

**Fig. 3:**
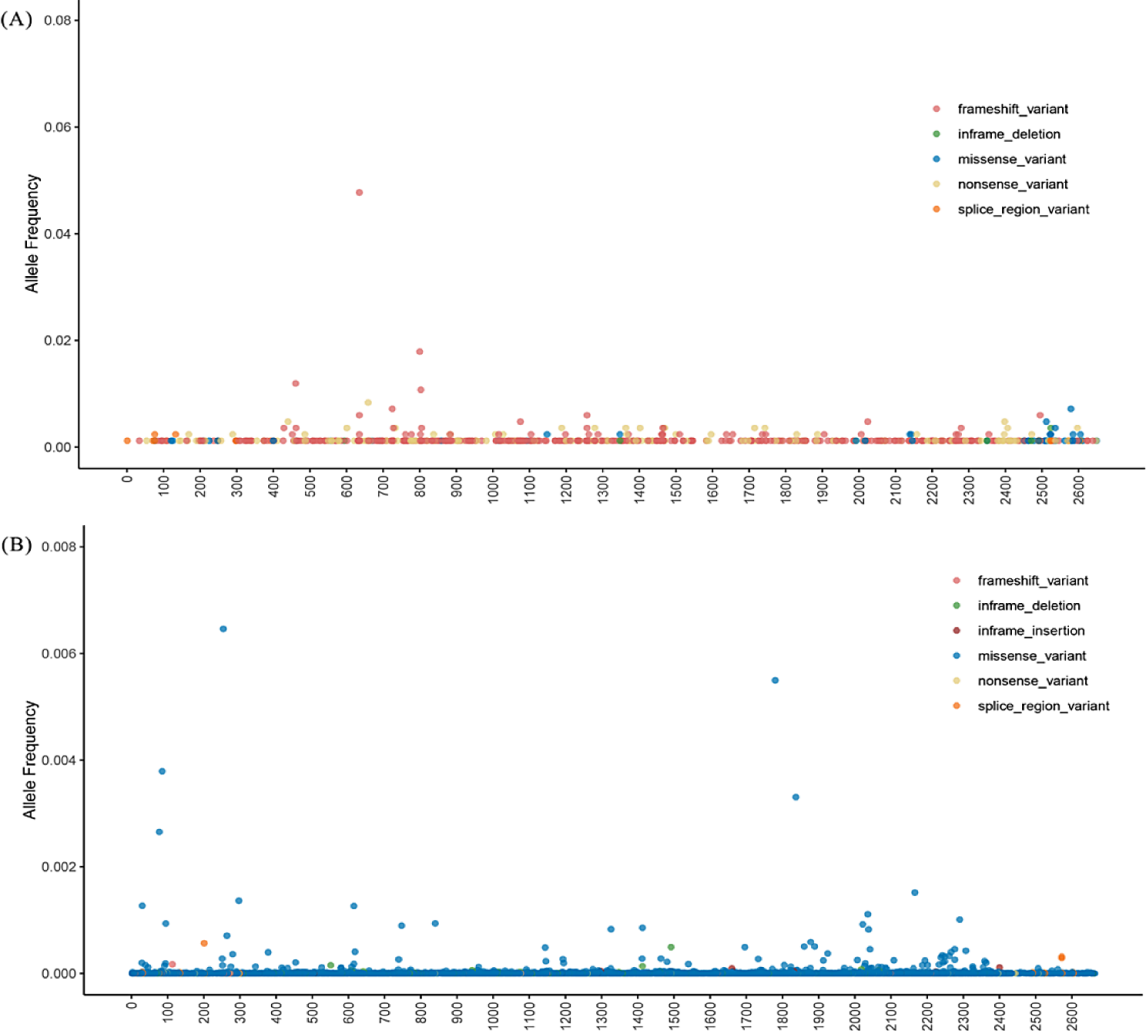
Frequency of *ANKRD11* variants in a total of 838 KBGS patients (**A**) and *ANKRD11* allele frequency in general population (**B**). *ANKRD11* allele frequency below 1% in general poulation was obtained from gnomAD (http://gnomad-sg.org/*).* The abscissa represents the full-length amino acid sequence of ANKRD11, and the ordinate represents the frequency

Mutation rates vary significantly along nucleotide sequences such that variants often concentrate at certain positions called hotspots [100]. DNA sequences prone to variation are highly dependent of gene sequence and structure as well as its chromosomal location, such as GC-rich region, microsatellites, meiotic recombination, nonallelic homologous recombination, centromeric rearrangements, telomeres and subtelomeric regions, replication timing and common fragile sites [101, 102]. Therefore, hotspot variants are indicative of the structural and functional properties of DNA sequence. Within the spectrum of *ANKRD11* variants, over two dozen distinct variants have been identified in at least three patients. Beyond a few variants that have been vertically inherited within a single family, the majority of variants were discovered in multiple sporadic patients, underscoring the propensity for these genetic variants to arise independently in unrelated individuals. Four hotspot variants of ANKRD11 protein were observed including p.Glu461Glnfs*48, p.Lys635Glnfs*26, p.Glu800Asnfs*62 and p.Lys803Argfs*5 (Fig. 3A). These four variants are frameshift variants generated by c.1381_1384delGAAA, c.1903_1907delAAACA, c.2395_2398delAAAG and c.2408_2412delAAAAA, respectively. Two additional prevalent frameshift variants were traced back to analogous genomic alterations including p.Asn725Lysfs*23 and p.Thr462Lysfs*47 arising from c.2175_2178delCAAA and c.1385_1388delCAAA, respectively. The propensity for short deletions within AAA-type-containing sequences may be associated with polymerase slippage events induced by tandem repeats, a well-established mechanism for indels [100]. Nonetheless, it should be highlighted that CCC-type-containing sequences exhibit a heightened vulnerability to this form of genetic variation [103, 104]. RD2 domain located at the C-terminus of ANKRD11 seemed to be particularly vulnerable to a range of variant events in KBGS patients, with missense variants being notably prevalent (Fig. 3A). Conversely, the missense variants occuring in RD2 domain were relatively rare in general population (Fig. 3B). This was consistent with the results of in vitro cellular assays, which showed that missense variants occurring in the RD2 domain impaired the protein function of ANKRD11 [66]. Some frameshift and nonsense variants of ANKRD11 have been identified in general population, such as p.Glu2082Argfs*20, p.Ser2180Phefs*6, p.Glu1075* and p.Gln2507*, indicating a pattern of variable expressivity and incomplete penetrance associated with *ANKRD11* variants [2]. Taken together, the presence of hotspot variants offers valuable insights into the inherent vulnerability of specific DNA sequence to abnormal DNA repair, replication, and modification or environmental exposures. These findings warrant in-depth exploration at the molecular level to unravel the underlying mechanisms and implications.

### *ANKRD11* variants and short stature in patients with KBGS

#### Frequency of occurrence of short stature in patients with *ANKRD11* variants

**Fig. 4:**
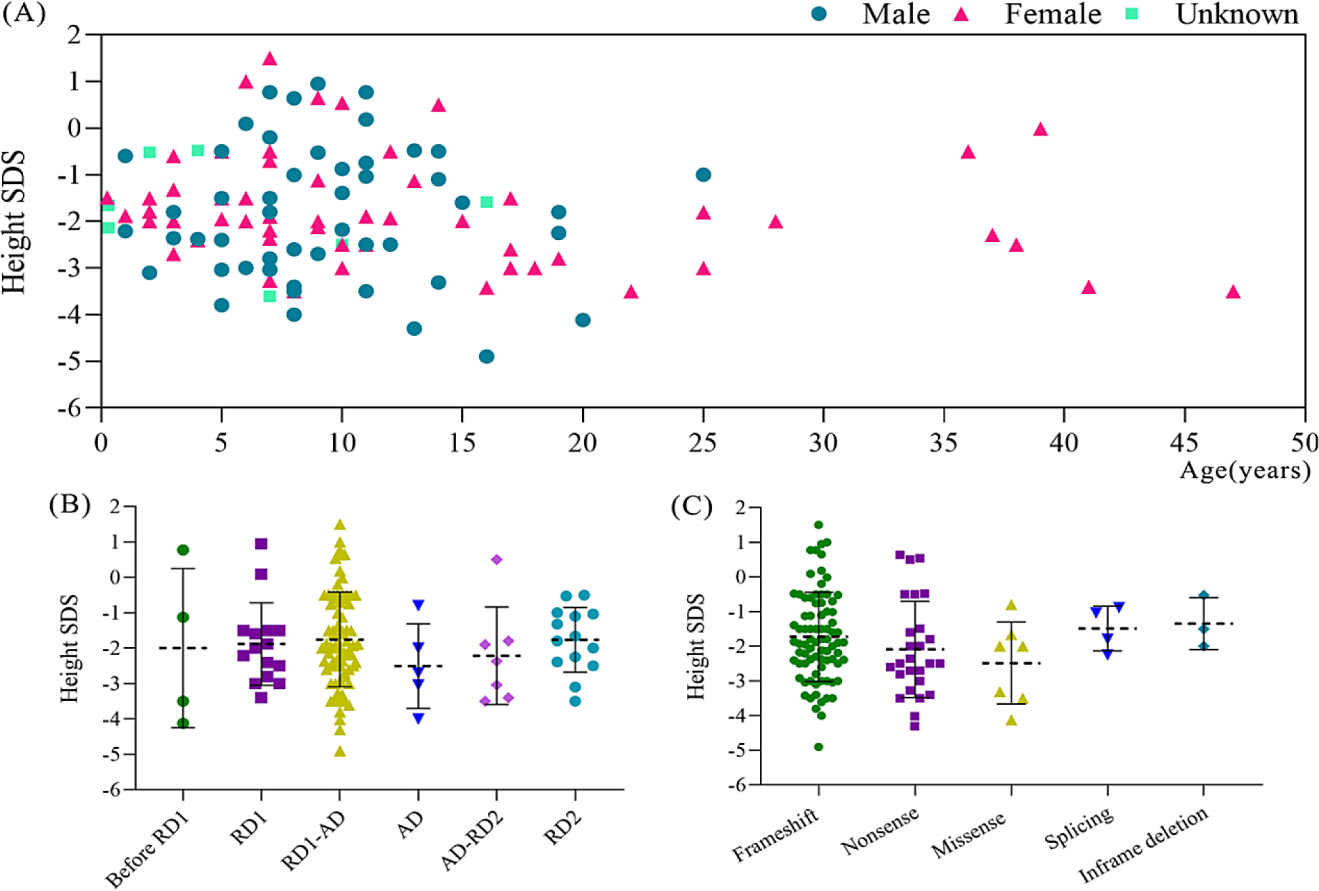
Distribution of gender and height SDS of patients having *ANKRD11* variants (**A**) and comparison of height SDS of patients having *ANKRD11* variants within different domain (**B**) or having different *ANKRD11* variant types (**C**). ANK: ankyrin repeat domain, RD1: repression domain 1, AD: activation domain, RD2: repression domain 2

Short stature is defined as height less than − 2 standard deviation (SD) or below the third percentile of corresponding mean height for age-, gender- and race-matched populations [105, 106]. As widely recognized, height is a highly heritable characteristic, and is classically influenced by hundreds of common variants pinpointed by genome-wide association studies (GWAS) [107, 108]. By comparison, the impact of rare and low-frequency monogenic variants on height is more pronounced, yielding a larger effect size compared to single nucleotide polymorphisms (SNPs) [109, 110]. Finding new genes with rare deleterious variants relating to growth is of considerable significance. Case series and individual reports serve as valuable sources of evidence for investigating the frequency of occurrence of short stature among patients harboring *ANKRD11* variants. In 121 patients reported with height SDS, a significant proportion, amounting to 48.76% (59/121), exhibited a height below the − 2 SDS (Fig. 4A). This prevalence was observed with nearly equal frequency across genders, with female patients exhibiting a rate of 46.43% (26/56) and male patients exhibiting a rate of 49.02% (25/51). The height SDS of females and males were − 1.80 ± 1.27 and − 1.85 ± 1.28 SDS, respectively. Upon incorporating additional patients recorded with height percentile values into the analysis, the proportion of patients with short stature was found to be 47.35% (116/245). Moreover, while some patients did not exhibit short stature, their adult height SDS or growth percentile might be lower than expected if their genetic potential (mid-parental height) was taken into account. However, most studies did not report patients’ genetic potential for height, making it challenging to extract this specific information from the published literature. Overall, approximately half of the patients with *ANKRD11* variants exhibited short stature, consequently, this characteristic stand as an important manifestation of KBGS attributable to *ANKRD11* variants. Certainly, compared to other features, the incidence of short stature was less frequent than that of craniofacial anomalies (100%), dental anomalies (80%) and intellectual disability (77%) [48]. Notably, patients with *ANKRD11* variants displayed a variable height phenotype ranging from as low as -4.9 SDS to as high as + 1.5 SDS. It can be ascribed to several factors, including genetic context of the gene, modified penetrance, variant type and variant location [111, 112]. There was no significant difference in height SDS among patients with *ANKRD11* variants located in different regions or with different ANKRD11 variant types (*p* > 0.05) (Fig. 4B&C). Previous investigation has revealed that terminations close to the C-terminus of the ANKRD11 protein tended to have less severe short stature, but the research did not yield a statistically significant difference or a clear trend in the severity of short stature among the various types of *ANKRD11* variants [39]. The findings of the current study indicated that no genotype-phenotype correlation was established. Certainly, a limited number of patients with *ANKRD11* variants across different domains present a significant constraint on this conclusion.

### Frequency of ANKRD11 pathogenic variants in short-stature cohorts

Functional variants in the *ANKRD11* gene have been identified through exome sequencing or gene panels in multiple short-stature cohorts (Table 1). The frequency of pathogenic variants was estimated to be between 0.35% and 0.55% [43, 68, 79, 113]. These variants were identified in patients initially diagnosed as having syndromic short stature, however, subsequent molecular diagnosis facilitated a more precise diagnosis of KBG syndrome. Syndromic short stature represents a phenotypic and genetically heterogeneous disease, and it accounts for a large part of the etiology of short stature. Considering the wide range of phenotypic manifestations and variable degree of severity, certain patients with short stature suffering from KBGS may not be accurately diagnosed in clinical practice. Consequently, it is likely that these patients harbor rare pathogenic variants in the *ANKRD11* gene, which may elude detection and result in their classification within the vast and enigmatic group of short stature with undetermined etiologies. Genetic testing should be factored into precise diagnosis of syndromic short stature in the future. Based on previous studies estimating the occurrence of short stature at approximately 3% [114–116], the prevalence of *ANKRD11* variants in the general population could be roughly calculated to be in the range of 0.0105–0.0165%. Nevertheless, given the limited sample sizes and the variability among different cohorts studied for short stature, the frequency of *ANKRD11* variants remains uncertain and requires a more accurate assessment. This evaluation should ideally be conducted through large-scale population screenings, employing artificial intelligence-enhanced phenotyping in conjunction with genetic testing [117]. Despite the growing awareness and attention this condition has recently garnered in the clinical and genetic research communities, there remains a significant gap in the identification and management of KBGS patients. Therefore, the development of international consensus guidelines for the diagnosis of KBGS is of paramount importance.

**Table 1:** Frequency of *ANKRD11* variants in multiple short-stature cohorts

No.	Region	Inclusion criteria	Number of subjects	Sequencing methods	Frequency	References
1	Brazil	SGA without catch-up growth and/or syndromic short stature	187 (M/F, unknown)	WES	0.00534759 (1/187)	[43]
2	China	IGHD; MPHD; GHI; SGA without catch-up growth; Height < -3SDS; Syndromic short stature	814 (M/F = 438/376)	330 by WES and 484 by inherited disease panel	0.004914 (4/814)	[68]
3	European	ISS; Syndromic short stature	200 (M/F = 78/122)	WES	0.005 (1/200)	[79]
4	China	ISS; Syndromic short stature	561 (M/F = 369/192)	WES	0.00356506 (2/561)	[113]

No.: number; SGA: small for gestational age; M: male; F: female; WES: whole exome sequencing; IGHD: isolated growth hormone deficiency; MPHD: multiple pituitary hormone deficiency; GHI: growth hormone insensitivity; ISS: idiopathic short stature

### Recombinant human growth hormone therapy

In 1985, recombinant human growth hormone (rhGH) received approval from the US Food and Drug Administration (FDA) for the treatment of children with severe GHD. Since then, over the past nearly forty years, the application of rhGH has been progressively expanded to enhance the height outcomes in children with a variety of growth disorders, including chronic renal insufficiency (CRI), ISS, SGA without catch-up growth, Prader-Willi Syndrome (PWS), Noonan syndrome (NS), Turner syndrome (TS) and SHOX haploinsufficiency [118, 119]. The advent of high-throughput sequencing technology has ushered in a period of rapid advancement in the field of genetics and genomics, and this progress has significantly broadened our capacity for diagnosing and treating conditions associated with short stature. We are now entering a transformative era characterized by molecular diagnosis and the tailoring of therapeutic interventions to the specific genetic makeup of individuals, including their responsiveness to rhGH therapy [120]. It has been observed that pathogenic variants in the aggrecan (*ACAN*), natriuretic peptide receptor 2 (*NPR2*), and Indian hedgehog (*IHH*) genes, which are integral to growth plate development, have been consistently associated with a positive response to rhGH therapy [121–125]. In this review, we delineated the growth response observed in patients harboring *ANKRD11* variants who received rhGH therapy (Table 2). The ages at initiation of rhGH treatment ranged from 5.2 to 14 years, and the treatment duration extended from 0.58 to 3 years. Following rhGH treatment, all patients exhibited varying levels of catch-up growth, as reflected by a range in Δ height SDS from 0.14 to 1.87. Among the nine patients, five showed a significant height improvement, reaching values above − 2 SDS ( -0.75 SDS for patient 3, − 0.7 SDS for patient 4, -1.86 SDS for patient 5, -1.8 SDS for patient 8 and − 1.91 SDS for patient 9). Most patients displayed either a good or moderate response to rhGH therapy. However, there was an exception with patient 3, a 7.9-year-old girl, whose height SDS only increased by 0.14 following a continuous treatment period of 0.58 years. Practically, a four-year-old girl form Australia with *ANKRD11* variant (c.6472G > T, p.Glu2158*), showed no response to rhGH therapy [49]. The girl was not included in Table 2 due to the lack of height data. The potential existence of additional factors that may be contributing to the suboptimal response to rhGH remains uncertain.

**Table 2:** Height outcomes of patients having ANKRD11 variants and receiving growth hormone treatment

No.	Gender	Country	ANKRD11 mutation	Initiation age of treatment (years)	HtSDS (before treatment)	HtSDS (after treatment)	Treatment time (years)	References
1	M	China	c.2579 C > T, p. S860L	14	-3.31	-2	2	[1]
2	F	China	c.6972dupC, p. P2271Pfs*8	7.9	-3.07	-2.93	0.58	[33]
3	M	Sweden	c.1903_1907delAAACA, p. K635Qfs*26	-	-2.5	-0.75	-	[42]
4	F	China	c.2635dupG, p. E879fs	5.5	-1.95	-0.7	2	[46]
5	M	Italy	c.7534 C > T, p. R2512W	11	-2.86	-1.86	1.5	[55]
6	F	Italy	c.3339G > A, p. W1113*	5.2	-2.92	-2.13	2.6	
7	M	Belgium	c.3836delG, p. S1279fs	10.5	-3.1	-2.5	1	[58]
8	M	Netherland	c.1903_1907delAAACA, p. K635Qfs*26	7.4	-2.8	-1.8	1	
9	M	South Korea	c.5889delC, p. I1963Mfs*9	6.5	-3.04	-1.91	3	[62]

No.: number, HtSDS: height SDS, F: female, M: male

Given the evidence suggesting that the *ANKRD11* gene acts as a potential tumor suppressor due to its interaction with the p53 protein, particular attention should be paid to the safety profile of rhGH therapy, particularly oncogenic risks [126]. However, observational studies have reported no increased risk of mortality or the development of primary cancers among pediatric patients receiving rhGH treatment [127–129]. The implementation of cancer surveillance in patients clinically diagnosed as having KBGS due to *ANKRD11* variants has been previously contemplated, and few patients were reported to develop malignant tumors [130, 131]. Short stature is one of all KBGS phenotypes that can be effectively treated with growth-promoting drugs, but there are few patients receiving rhGH treatment. The approval and accessibility of rhGH therapy for KBGS may be limited in certain countries, which highlights the imperative for further investigation and research within this specialized domain. In alignment with the recommendations proposed by Reynaert et al. [58], we advocate for a more favorable stance towards the implementation of short-term rhGH therapy for *ANKRD11* variant-induced KBGS patients with severe short stature.

### Underlying mechanisms of *ANKRD11* variants causing short stature

**Fig. 5:**
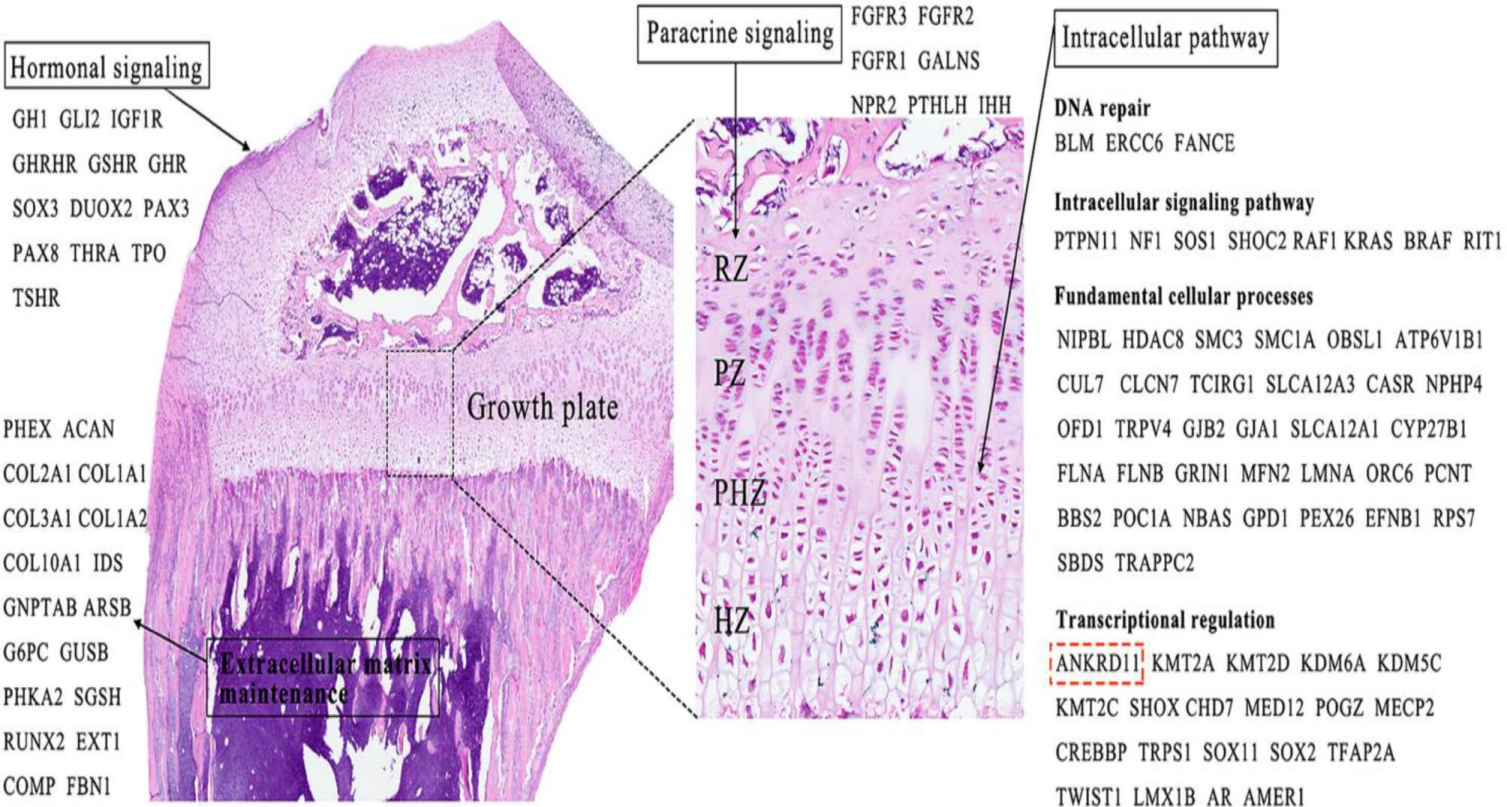
Disease-causing genes associated with short stature through affecting the endochondral ossification of epiphyseal growth plate. The *ANKRD11* gene may be implicated in this process as a transcription regulator. RZ: resting zone, PZ: proliferative zone, PHZ: prehypertrophic zone, HZ: hypertrophic zone

Human longitudinal bone growth is persistently driven by the process of endochondral ossification within the epiphyseal growth plate that is characterized by three histologically distinct zones (resting, proliferative, and hypertrophic zones) throughout the stages of postnatal development [132]. As the slowly-cycling reserve cells, resting chondrocytes are maintained in a wingless-related integration site (Wnt)-inhibitory environment, and it contains a certain proportion of parathyroid hormone-related protein (PTHrP)-expressing skeletal stem-like cells producing rapidly proliferating columnar chondrocytes parallel to the direction of bone elongation [133]. Proliferative zone chondrocytes will differentiate into hypertrophic chondrocytes characterized by specific expression of type X collagen gene (*Col10a1*), and further undergo apoptosis or osteoblasts trans-differentiation, thereby contributing to bone elongation [134, 135]. The orchestrated differentiation of chondrocytes within the growth plate is governed by a complex interplay of numerous genes that are involved in a variety of signaling pathways, including hormonal signaling, paracrine signaling, intracellular pathways and extracellular matrix homeostasis (Fig. 5) [68, 136–138]. Functional variants in any of these genes can disrupt the growth plate chondrogenesis and impair the subsequent bone elongation. It was hypothesized that ANKRD11 plays a direct role in the transcriptional regulation of certain critical genes via intracellular pathways in the process of growth plate development [68]. In a prior investigation, Yoda mice with an N-ethyl-N-nitrosourea (ENU)-induced mutation in the *ANKRD11* gene, exhibited a markedly reduced body size and presented with a phenotype reminiscent of osteoporosis compared to littermate controls [3]. However, no alterations were observed in the histological structure of the tibial growth plate and plasma IGF-1 level between six-month-old Yoda mice and wild-type mice. Given that growth plate in rodents do not undergo fusion but are instead subject to an age-related decrease following sexual maturation [139], it can be inferred that adult mice with *ANKRD11* deficiency may not well accurately reflect the aberrant differentiation process of growth plate chondrocytes during rapid bone elongation. Data obtained from the International Mouse Phenotyping Consortium (IMPC) indicate that C57BL/6 N mice carrying a heterozygous *ANKRD11*^tm1b(EUCOMM)Wtsi^ allele exhibited a reduction in body length when compared to their littermate controls (https://www.mousephenotype.org/data/genes/MGI:1924337*).* Additionally, mice with a conditional deletion of the *ANKRD11* gene in neural crest cells dispalyed ossification centers that were either incapable of expansion or failed to fuse, demonstrating the critical regulatory role of *ANKRD11* gene in intramembranous ossification [22]. In vitro studies further revealed that ANKRD11 was capable of enhancing the transactivation of the *p21* gene, a key factor in the chondrogenic differentiation of ATDC5 cells induced by insulin supplements [61]. The chondrogenic differentiation of ATDC5 cells induced by insulin-transferrin-selenium is a widely recognized in vitro model mimicking endochondral ossification [140–143]. The potential role of the ANKRD11-p21 signaling pathway in growth plate development as a plausible mechanism to elucidate the short stature observed in KBGS patients warrants further investigation. To elucidate the functional mechanisms of the *ANKRD11* gene in the physiological process of growth plate development, it is essential to conduct further study employing a mouse model with chondrocyte-specific ANKRD11 ablation, utilizing the CRISPR/Cas9 and Cre/LoxP recombination system.

## Conclusions

Frameshift and nonsense were the most common types of *ANKRD11* variants. Approximately half of the KBGS patients harboring ANKRD11 variants had short stature. However, the current study has not established a clear correlation between the genotype and this phenotypic manifestation. Some patients harboring *ANKRD11* variants may initially be diagnosed as syndromic short stature due to limited recognition of KBGS. While patients with *ANKRD11* variants exhibit a positive response to rhGH therapy, further investigation is warranted to substantiate its efficacy and safety. Functional variants in the *ANKRD11* gene can potentially disrupt the longitudinal growth of bones by influencing the orderly differentiation process of growth plate chondrocytes, which needs deeper investigation through fundamental research to elucidate its underlying mechanisms.

### Electronic supplementary material

Below is the link to the electronic supplementary material.

Supplementary Material 1

Supplementary Material 2

Supplementary Material 3

## Data Availability

The original contributions presented in the study are included in the article/Supplementary Materials, further inquiries can be directed to the corresponding authors.

## Abbreviations

ACAN: Aggrecan
ACMG: American college of medical genetics and genomics
AD: Activation domain
ADA3: Alteration/deficiency in activation 3
AIB1: Amplified in breast cancer 1
AMP: Association for molecular pathology
ANCO: Ankyrin repeats-containing cofactor
ANK: Ankyrin
ANKRD11: Ankyrin repeat domain containing-protein 11
ANOVA: One-way analysis of variance
BDNF: Brain-derived neurotrophic factor
CAF: [(CAMP-response-element binding protein)-binding protein]-associated factor
CBP: (CAMP-response-element binding protein)-binding protein
CREB: CAMP-response-element binding protein
CRI: Chronic renal insufficiency
EEG: Electroencephalogram
ENU: N-ethyl-N-nitrosourea
ER: Estrogen receptor
FDA: Food and drug administration
GH: Growth hormone
GHD: Growth hormone deficiency
GHI: Growth hormone insensitivity
GWAS: Genome-wide association study
HAT: Histone acetylase
HDAC: Histone deacetylase
H3K9: Histone 3 lysine 9
H4K5: Histone 4 lysine 5
H4K8: Histone 4 lysine 8
H4K16: Histone 4 lysine 16
HtSDS: Height standard deviation score
HZ: Hypertrophic zone
IGF-1: Insulin-like growth factor
IGFBP-3: Insulin-like growth factor binding protein 3
IGHD: Isolated growth hormone deficiency
IHH: Indian hedgehog
IMPC: International mouse phenotyping onsortium
Indel: Insertion or deletion
IQ: Intelligence quotient
ISS: Idiopathic short stature
LBD: Ligand-binding domain
MPHD: Multiple pituitary hormone deficiency
MRI: Magnetic resonance imaging
NLS: Nuclear localization signal
NPR2: Natriuretic peptide receptor 2
NRs: Nuclear receptors
NS: Noonan syndrome
OMIM: Online mendelian inheritance in man
PAS: Per-Arnt-Sim
PTHrP: Parathyroid hormone-related protein
PTV: Protein-truncating variant
PWS: Prader-Willi syndrome
PZ: Proliferative zone
RD: Repression domain
rhGH: Recombinant human growth hormone
RZ: Resting zone
SD: Standard deviation
SDS: Standard deviation score
SGA: Small for gestational age
SNP: Single nucleotide polymorphism
SOC: Secondary ossification center
TEAD: Transcriptional enhanced associate domain
TrkB: Tyrosine receptor kinase B
TS: Turner syndrome
WES: Whole exome sequencing
Wnt: Wingless-related integration site
YAP: Yes-associated protein
