# Supplementary material for: Insights into the ANKRD11 variants and short-stature phenotype through literature review and ClinVar database search
Source: Orphanet J Rare Dis. 2024 Aug 12;19:292. doi: 10.1186/s13023-024-03301-y (PMC11318275; doi:10.1186/s13023-024-03301-y)
Supplement: Supplementary file 2 — Supplementary Material 2 [file 13023_2024_3301_MOESM2_ESM.docx]

**Title:** Insights into the ANKRD11 variants and short-stature phenotype through literature review and ClinVar database search

**Journal:** Orphanet Journal of Rare Diseases

**Authors:** Dongye He, Mei Zhang, Yanying Li, Fupeng Liu, Bo Ban

**Correspondence:** Dongye He ([hehe0917@mail.jnmc.edu.cn](mailto:hehe0917@mail.jnmc.edu.cn)); Bo Ban ([banbo2011@163.com](mailto:banbo2011@163.com)), Department of Endocrinology, Genetics and Metabolism and Medical Research Center, Affiliated Hospital of Jining Medical University, 89 Guhuai Road, Jining 272029, Shandong, China.

**Caption patients having *ANKRD11* variants from published literature and ClinVar database.**

| **Patients reported in literature** | | | | | | | |
| --- | --- | --- | --- | --- | --- | --- | --- |
| No. | Gender | Age | ANKRD11 variants | Birth weight | Birth height | Height SDS | Reference |
| 1 | M | 14 | c.2579C>T, p.S860L | -0.3 | -0.2 | -3.31 | [1] |
| 2 | F | 13 | c.915delA, p.Pro306Hisfs*62 | 0.53 | 0.42 | -1.13 | [2] |
| 3 | M | 6 | c.1711_1723del, p.Thr571Alafs*15 | -0.34 | -0.04 | 0.09 |  |
| 4 | F | 7 | c.1977C>A, p.Tyr659* | -0.54 | -1.7 | -3.27 |  |
| 5 | F | 9 | c.2398_2401delGAAA, p.Glu800Asnfs*62 | -1.83 | - | -2.12 |  |
| 6 | F | 17 | c.2408_2412delAAAAA, p.Lys803Argfs*5 | 0.87 | -0.32 | -2.6 |  |
| 7 | F | 10 | c.2692C>T, p.Arg898* | -1.78 | 0.63 | 0.54 |  |
| 8 | F | 3 | c.7356dupC, p.Lys2453Glnfs*79 | -0.56 | -0.15 | -1.32 |  |
| 9 | M | 9 | c.7411_7422del, p.Thr2471_Gly2474del | 0.08 | 1.64 | -0.53 |  |
| 10 | M | 10 | c.1903_1907delAAACA, p.Lys635Glnfs*26 | -1.75 | -1.95 | -1.39 |  |
| 11 | M | 8 | c.4218C>A, p.Tyr1406* | -1.94 | -1.65 | 0.64 |  |
| 12 | M | 3 | c.4087C>T, p.Arg1363* | -2.5 | -0.92 | -2.36 |  |
| 13 | M | 11 | c.7470 + 2 T>C | -0.18 | - | -1.04 |  |
| 14 | M | 1 | c.1381_1384delGAAA, p.Glu461Glnfs*48 | -1.65 | -1.39 | -2.21 |  |
| 15 | F | 11 | c.1903_1907delAAACA, p.Lys635Glnfs*26 | 1.38 | 1.94 | -1.89 |  |
| 16 | F | 9 | c.3888dupC, p.Asn1297Glnfs*3 | -1.36 | -1.23 | -1.12 |  |
| 17 | M | 13 | c.3591_3594del, p.Lys1198Argfs*119 | -1.98 | -2.23 | -0.48 |  |
| 18 | M | 9 | c.1381_1384delGAAA, p.Glu461Glnfs*48 | 2.72 | 3.45 | 0.95 |  |
| 19 | M | 7 | c.1903_1907delAAACA, p.Lys635Glnfs*26 | -0.74 | 0.22 | 0.77 |  |
| 20 | F | 12 | c.1903_1907delAAACA, p.Lys635Glnfs*26 | 0.86 | 0.09 | -1.93 |  |
| 21 | M | 8 | c.2408_2412delAAAAA, p.Lys803Argfs*5 | 0.31 | - | -1.01 |  |
| 22 | F | 15 | c.5123C>A, p.Ser1708* | -2.26 | - | -1.99 |  |
| 23 | F | 4 | c.1381_1384delGAAA, p.Glu461Glnfs*48 | - | - | -2.4 |  |
| 24 | M | 25 | p.Glu2524_Lys2525del | - | - | 3^rd^ | [15] |
| 25 | M | 22 | p.Glu2524_Lys2525del | - | - | <3^rd^ |  |
| 26 | M | 46 | p.Glu2524_Lys2525del | - | - | <3^rd^ |  |
| 27 | M | 9 | c.2305delT, p.Ser769Glnfs*8 | - | - | <3^rd^ |  |
| 28 | M | 6 | c.7189C>T, p.Gln2397* | - | - | >3^rd^ |  |
| 29 | M | 21 | c.5953_5954delCA, p.Gln1985Glufs*46 | - | - | <3^rd^ |  |
| 30 | M | 9 | c.6071_6084delCGTACGCTCTGCCC, p.Pro2024Argfs*3 | - | - | <3^rd^ |  |
| 31 | M | - | c.3224_3227del, p.Glu1075Glyfs*242 | - | - | >3^rd^ | [17] |
| 32 | F | 7 | c.1903_1907del, p.Lys635Glnfs*26 | - | - | <3^rd^ | [18] |
| 33 | M | 10 | c.3224_3227del, p.Glu1075Glyfs*242 | - | - | >3^rd^ |  |
| 34 | F | 2 | c.6187G>T, p.Glu2063* | - | - | >3^rd^ |  |
| 35 | M | 7 | c.6416C>T, p.Pro2139Leu | - | - | <3^rd^ |  |
| 36 | F | 30 | c.6416C>T, p.Pro2139Leu | - | - | <3^rd^ |  |
| 37 | F | 15 | c.1785_1786delGCinsTT, p.R595_A2663delinsS | - | - | <3^rd^ | [19] |
| 38 | M | 13 | c.1903_1907delAAACA, p.K635Qfs*26 | - | - | <3^rd^ |  |
| 39 | M | 26 | c.2130delG, p.W710Cfs*9 | - | - | >3^rd^ |  |
| 40 | M | 15 | c.4283_4286delAAGA, p.K1428Ifs*13 | - | - | <3^rd^ |  |
| 41 | M | 15 | c.6817_6833delGGCCCCGCCCCGAACAC, p.G2273Cfs*17 | - | - | <3^rd^ |  |
| 42 | M | 13 | c.7535G>A, p.R2512Q | - | - | >3^rd^ |  |
| 43 | M | 20 | c.362T>A, p.Met121Lys | 3500 g | - | -4.12 | [25] |
| 44 | F | - | c.7571A>G, p.Glu2524Gly | - | - | - | [26] |
| 45 | F | - | c.7571A>G, p.Glu2524Gly | - | - | - |  |
| 46 | F | 2 | c.1366_1367dup, p.K457Rfs*54 | -2.5 | -2.2 | -2.0 | [27] |
| 47 | M | 9 months | c.2395-2398delAAAG, p.Glu800Asnfs*62 | <3^rd^ | <3^rd^ | <3^rd^ | [28] |
| 48 | F | 21 months | c.2395-2398delAAAG, p.Glu800Asnfs*62 | <3^rd^ | <3^rd^ | <3^rd^ |  |
| 49 | F | 37 | c.2395-2398delAAAG, p.Glu800Asnfs*62 | - | - | <3^rd^ |  |
| 50 | M | 19 | c.7471-1G>C | - | - | -2.25 | [29] |
| 51 | F | 18 | c.1903_1907del,p.Lys635Glnfs*26 | -2.40 | - | -3.00 | [30] |
| 52 | M | 11 | c.4171C>T, p.Gln1391* | - | - | <3^rd^ | [31] |
| 53 | M | 12 | c.1903_1907del, p.Lys635Glnfs*26 | - | - | <3^rd^ |  |
| 54 | M | 12 | c.3931C>T, p.Arg1311* | - | - | 85^th^ |  |
| 55 | F | 4 | c.2709del, p.Phe904fs | - | - | - | [32] |
| 56 | M | 7 | c.6972dupC, p.P2271Pfs*8 | 2400g | 48cm | -3.04 | [33] |
| 57 | F | 7 | c.2408_2412delAAAAA, p.Lys803Argfs*5 | 2740g | 48cm | <3^rd^ | [34] |
| 58 | F | 4 | c.5483G>T, p.S1828* | -1.6 | 1.75 | <3^rd^ | [35] |
| 59 | M | 15 | c.2297_2300delAGAA, p.K766_K767fs*9 | -1.7 | 0.3 | -1.6 |  |
| 60 | M | 13 | c.5117delC, p.Pro1706Leufs*13 | 4060g | 57cm | <3^rd^ | [36] |
| 61 | F | 6 | c.2408_2412del, p.Lys803Argfs*5 | - | - | <0.4^th^ | [37] |
| 62 | M | 10 | c.2398_2401del, p.Glu800Asnfs*62 | - | - | >3^rd^ |  |
| 63 | F | 4 | c.5426_5430del, p.Leu1809Glnfs*139 | - | - | >3^rd^ |  |
| 64 | M | 13 | c.1903_1907del, p.Lys635Glnfs*26 | - | - | >3^rd^ |  |
| 65 | F | 21 | c.1903_1907del, p.Lys635Glnfs*26 | - | - | <3^rd^ |  |
| 66 | F | 19 | c.1903_1907del, p.Lys635Glnfs*26 | - | - | <3^rd^ |  |
| 67 | F | 12 | c.1903_1907del, p.Lys635Glnfs*26 | - | - | >3^rd^ |  |
| 68 | M | 47 | c.1903_1907del, p.Lys635Glnfs*26 | - | - | >3^rd^ |  |
| 69 | M | 23 | c.5274dup, p.Ser1759Leufs*38 | - | - | >3^rd^ |  |
| 70 | F | 11 | c.3437_3461del, p.Thr1146Lysfs*164 | - | - | <3^rd^ |  |
| 71 | M | 3 | c.1903_1907del, p.Lys635Glnfs*26 | - | - | >3^rd^ |  |
| 72 | M | 17 | c.1381_1384del, p.Glu461Glnfs*48 | - | - | >3^rd^ |  |
| 73 | F | 17 | c.1801C>T, p.Arg601* | - | - | >3^rd^ |  |
| 74 | F | 10 | c.4103_4104del, p.Lys1368Argfs*17 | - | - | >3^rd^ |  |
| 75 | F | 12 | c.3208_3209del, p.His1070Trpfs*31 | - | - | >3^rd^ |  |
| 76 | M | 6 | c.4177_4189del, p.Glu1393Trpfs*12 | - | - | >3^rd^ |  |
| 77 | M | 6 | c.2408_2412del, p.Lys803Argfs*5 | - | - | <3^rd^ |  |
| 78 | M | 9 | c.1903_1907del, p.Lys635Glnfs*26 | - | - | <3^rd^ |  |
| 79 | F | 13 | c.2408_2412del, p.Lys803Argfs*5 | - | - | >3^rd^ |  |
| 80 | F | 10 | c.3582del, p.Arg1195Glufs*123 | - | - | >3^rd^ |  |
| 81 | M | 8 | c.3704_3707del, p.Lys1235Argfs*82 | - | - | >3^rd^ |  |
| 82 | M | 10 | c.4206C>G, p.Tyr1402* | - | - | >3^rd^ |  |
| 83 | M | 10 | c.5199_5227del, p.Asp1734Alafs*53 | - | - | >3^rd^ |  |
| 84 | M | 10 | c.6364_6367del, p.Asp2122Pro*52 | - | - | >3^rd^ |  |
| 85 | F | 9 | c.1903_1907del, p.Lys635Glnfs*26 | - | - | >3^rd^ |  |
| 86 | M | 23 | c.1801C>T, p.Arg601* | - | - | <3^rd^ |  |
| 87 | F | 2 | c.4408A>T, p.Arg1470* | - | - | <3^rd^ |  |
| 88 | M | 21 | c.2182_2183del, p.Ile728Glnfs*13 | - | - | >3^rd^ |  |
| 89 | F | 46 | c.2182_2183del, p.Ile728Glnfs*13 | - | - | >3^rd^ |  |
| 90 | F | 15 | c.2182_2183del, p.Ile728Glnfs*13 | - | - | >3^rd^ |  |
| 91 | F | 6 | c.2512C>T, p.Arg838* | - | - | >3^rd^ |  |
| 92 | F | 22 | c.867C>G, p.Tyr289* | - | 25th | -3.5 | [38] |
| 93 | F | 10 | c.1120G>T, p.Glu374* | - | <1st | -2.5 |  |
| 94 | F | 5 | c.1367_1370del, p.Lys456Serfs*53 | - | 12th | -1.5 |  |
| 95 | F | 17 | c.1381_1384del, p.Glu461Glnfs*48 | - | 12th | -1.5 |  |
| 96 | F | 2 | c.1381_1384del, p.Glu461Glnfs*48 | - | - | -1.5 |  |
| 97 | F | 25 | c.1381_1384del, p.Glu461Glnfs*48 | - | 50th | -3 |  |
| 98 | M | 14 | c.1893dup, p.His632Thrfs*2 | - | <1st | -0.5 |  |
| 99 | F | 11 | c.1903_1907del, p.Lys635fs*26 | - | 7th | -2.5 |  |
| 100 | F | 7 | c.1903_1907del, p.Lys635fs*26 | - | 5th | 1.5 |  |
| 101 | M | 5 | c.2398_2401del, p.Glu800Asnfs*62 | - | <1st | -1.5 |  |
| 102 | F | 17 | c.2647G>T, p.Glu883* | - | 42nd | -3 |  |
| 103 | F | 7 | c.3045del, p.Asp1016Ilefs*302 | - | <1st | -0.5 |  |
| 104 | F | 6 | c.3153del, p.Glu1051Aspfs*267 | - | 70th | 1 |  |
| 105 | F | 5 | c.3198_3199del, p.His1066Glnfs*35 | - | 50th | -0.5 |  |
| 106 | M | 8 | c.3774_3775del, p.Lys1259Valfs*23 | - | <1st | -3.5 |  |
| 107 | M | 11 | c.4087C>T, p.Arg1363* | - | 67th | -2.5 |  |
| 108 | M | 5 | c.4786G>T, p.Glu1596* | - | 2nd | -0.5 |  |
| 109 | F | 14 | c.6766C>T, p.Gln2256* | - | 2nd | 0.5 |  |
| 110 | F | 8 | c.6792dup, p.Ala2265Argfs*8 | - | 12th | -3.5 |  |
| 111 | M | 8 | c.6792dupC, p.P2271Pfs*8 | 2400g | 48cm | -3.4 | [39] |
| 112 | M | 2 | c.7407dupC, p.P2530Rfs*61 | 2600g | 48cm | -3.1 |  |
| 113 | M | 8 | c.G3046A, p.D1016N | 3000g | 50cm | -3.5 |  |
| 114 | - | - | c.3255_3256del, p.Lys1086Glufs*15 | - | - | - | [40] |
| 115 | - | - | c.5434C>T, p.Gln1812* | - | - | - |  |
| 116 | M | 11 | C.1903_1907del, p.Lys635Glnfs*26 | -1.95 | -0.50 | 0.18 | [41] |
| 117 | M | 25 | c.7481dup, p.Pro2495fs | - | - | -1 | [42] |
| 118 | M | 14 | c.7481dup, p.Pro2495fs | - | - | -1.1 |  |
| 119 | F | 12 | c.7481dup, p.Pro2495fs | - | - | -0.5 |  |
| 120 | M | 7 | c.7481dup, p.Pro2495fs | - | - | -1.8 |  |
| 121 | F | 47 | c.7481dup, p.Pro2495fs | - | - | -3.5 |  |
| 122 | M | 12 | c.4391_4392del, p.Lys1464fs | - | - | -2.5 |  |
| 123 | M | 8 | c.6184del, p.Leu2062fs | - | - | -4 |  |
| 124 | F | 25 | c.3123_3126del, p.Ile1042fs | - | - | -1.8 |  |
| 125 | M | 6 | c.1460_1463del, p.Glu487fs | - | - | -3 |  |
| 126 | F | 38 | c.1903_1907del, p.Lys635fs | - | - | -2.5 |  |
| 127 | M | 9 | c.3832A>T, p.Lys1278* | - | - | -2.7 |  |
| 128 | F | 36 | c.3832A>T, p.Lys1278* | - | - | -0.5 |  |
| 129 | F | 28 | c.3832A>T, p.Lys1278* | - | - | -2 |  |
| 130 | M | 11 | c.2751dup, p.Glu918* | - | - | -3.5 |  |
| 131 | F | 10 | c.3382_3383del, p.Asp1128fs | - | - | -3 |  |
| 132 | M | 11 | c.1903_1907del, p.Lys635fs | - | - | -0.75 |  |
| 133 | M | 19 | c.6513dup, p.Gly2172fs | - | - | -1.8 |  |
| 134 | F | 19 | c.1318C>T, p.Arg440* | - | - | -2.8 |  |
| 135 | F | 41 | c.1318C>T, p.Arg440* | - | - | -3.4 |  |
| 136 | M | 16 | c.2398_2401del, p.Glu800Asnfs*62 | -1.7 | -2.0 | -4.9 | [43] |
| 137 | - | - | p.L876Pfs*6 | - | - | - | [44] |
| 138 | - | - | p.K2070Nfs*31 | - | - | - |  |
| 139 | - | - | c.4388delGAGA, p.K1464fs | - | - | - | [45] |
| 140 | F | 5 | c.2635dupG, p.Glu879fs | 0.58 | 0.1 | -1.95 | [46] |
| 141 | - | - | c.6071_6084del14, p.Pro2024Argfs*3 | - | - | - | [47] |
| 142 | - | - | c.5712_5713insT, p.Gly1905Trpfs*45 | - | - | - |  |
| 143 | - | - | c.5144dupA, p.Tyr1715* | - | - | - |  |
| 144 | - | - | c.3591_3594delAAAA, p.Lys1198Argfs*119 | - | - | - |  |
| 145 | - | - | c.7567C>T, p.Arg2523Trp | - | - | - |  |
| 146 | M | 3 | c.3562C>T, p.R1188* | - | - | -1.8 | [48] |
| 147 | M | 1 | c.2398_2401del, p.E800Nfs*62 | - | - | -0.6 |  |
| 148 | M | 5 | c.4911delT, p.P1638Lfs*48 | - | - | -3.8 |  |
| 149 | F | 3 | c.5659C>T, p.Q1887* | - | - | -2.7 |  |
| 150 | M | 15 | c.1801C>T, p.R601* | - | - | -1.6 |  |
| 151 | M | 7 | c.1903_1907del, p.K635Qfs*26 | - | - | -0.2 |  |
| 152 | F | 7 | c.1903_1907del, p.K635Qfs*26 | - | - | -0.7 |  |
| 153 | F | 3 | c.1903_1907del, p.K635Qfs*26 | - | - | -0.6 |  |
| 154 | F | 6 | c.2262dupA, p.E755Rfs*27 | - | - | -1.5 |  |
| 155 | F | 3 | c.7832A>T, p.H2611L | - | - | -2 |  |
| 156 | F | 7 | c.6528_6538del, p.G2177Hfs*5 | - | - | -1.9 |  |
| 157 | M | - | C.1903_1907del, p.Lys635Glnfs*26 | 10-50th | 50^th^ | 25^th^ | [49] |
| 158 | M | - | C.1903_1907del, p.Lys635Glnfs*26 | 50-90th | - | 3^rd^ |  |
| 159 | F | - | C.1903_1907del, p.Lys635Glnfs*26 | - | - | 50^th^ |  |
| 160 | F | - | c.505G>T, p.Glu169* | 50th | 50-90^th^ | 25-50^th^ |  |
| 161 | F | - | c.4406G>A, p.Trp1469* | <10th | <10^th^ | 75^th^ |  |
| 162 | F | - | c.4406G>A, p.Trp1469* | - | - | 3^rd^ |  |
| 163 | F | - | c.4406G>A, p.Trp1469* | - | - | 3^rd^ |  |
| 164 | F | - | C.1903_1907del, p.Lys635Glnfs*26 | 10-50^th^ | - | 1^st^ |  |
| 165 | M | - | c.1173C>G, p.Tyr391* | 10-50^th^ | 50-90^th^ | 25^th^ |  |
| 166 | F | 28 | c.7471A>C, p.Ile2491Leu | 10^th^ | - | 10-25^th^ |  |
| 167 | F | 34 | c.6409_6410del, p.Ser2137Profs*9 | - | - | <3^rd^ |  |
| 168 | F | 4 | c.3224_3227delCTTT, p.Glu1075Glyfs*242 | 10th | 10th | 10^th^ |  |
| 169 | M | 1 | c.7216C>T, p.Gln2406* | 25th | - | 10^th^ |  |
| 170 | F | 6 | c.3422G>A, p.Gly1148Ser | <10th | - | 10-25^th^ |  |
| 171 | M | 33 | c.3442G>A, p.Gly1148Ser | - | - | 75-90^th^ |  |
| 172 | F | 4 | c.6472G>T, p.Glu2158* | 90th | - | <3^rd^ |  |
| 173 | F | - | c.1285_1286delTC, p.Ser429Glyfs*8 | - | - | - | [50] |
| 174 | F | - | c.1385_1388delCAAA, p.Thr462Lysfs*47 | - | - | - |  |
| 175 | F | 2 | c.1457C>G, p.Ser486* | - | - | >3^rd^ |  |
| 176 | F | 8 | c.1903_1907delAAACA, p.Lys635Glnfs*26 | - | - | - |  |
| 177 | M | - | c.1977C>G, p.Tyr659* | - | - | - |  |
| 178 | M | 3 | c.1977C>G, p.Tyr659* | - | - | >3^rd^ |  |
| 179 | M | - | c.2175_2178delCAAA, p.Asn725Lysfs*23 | - | - | - |  |
| 180 | F | - | c.2175_2178delCAAA, p.Asn725Lysfs*23 | - | - | - |  |
| 181 | F | 15 | c.2197C>T, p.Arg733* | - | - | - |  |
| 182 | M | - | c.2398_2401delGAAA, p.Glu800Asnfs*62 | - | - | - |  |
| 183 | F | 8 | c.2408_2412delAAAA, p.Lys803Argfs*5 | - | - | - |  |
| 184 | F | 14 | c.2412delA, p.Glu805Lysfs*58 | - | - | - |  |
| 185 | F | - | c.3019C>T, p.Arg1007* | - | - | - |  |
| 186 | F | 3 | c.3180dupA, p.Asp1061Argfs*7 | - | - | >3^rd^ |  |
| 187 | F | 3 | c.3309dupA, p.Asp1104Argfs*2 | - | - | <3^rd^ |  |
| 188 | F | - | c.3770_3771delAA, p.Lys1257fs*25 | - | - | - |  |
| 189 | F | 1 | c.4107_4108delGA, p.Lys1370Glyfs*15 | - | - | >3rd |  |
| 190 | M | 8 | c.4374_4375delGA, p.Lys1459Glufs*94 | - | - | - |  |
| 191 | F | - | c.4389_4390delGA, p.Lys1464Thrfs*89 | - | - | - |  |
| 192 | M | - | c.4498C>T, p.Gln1500* | - | - | - |  |
| 193 | F | - | c.5146G>T, p.Glu1716* | - | - | - |  |
| 194 | F | 10 | c.5205delC, p.Val1736Cysfs*227 | - | - | - |  |
| 195 | F | - | c.5712_5713insT, p.Gly1905Trpfs*45 | - | - | - |  |
| 196 | F | - | c.6513dupC, p.Gly2172Argfs*14 | - | - | - |  |
| 197 | F | - | c.7000C>T, p.Gln2334* | - | - | - |  |
| 198 | M | - | c.7192C>T, p.Gln2398* | - | - | - |  |
| 199 | M | - | c.7216C>T, p.Gln2406* | - | - | - |  |
| 200 | M | - | c.7416C>G, p.Tyr2472* | - | - | - |  |
| 201 | M | 8 | c.5488G>T, p.E1830* | - | - | -2.6 | [51] |
| 202 | M | 13 | c.5488G>T, p.E1830* | - | - | -4.3 |  |
| 203 | M | 13 | c.6015dupA, p.Gly2006Argfs*26 | - | - | 10^th^ | [52] |
| 204 | F | 10 | c.3045del, p.Asp1016Ilefs*302 | - | - | 10^th^ | [53] |
| 205 | F | 14 | c.5145C>G, p.Tyr1715* | 2971g | 33cm | - | [54] |
| 206 | F | 13 | c.5145C>G, p.Tyr1715* | - | - | - |  |
| 207 | M | - | c.2194C>T, p.Arg733* | - | - | <3^rd^ | [55] |
| 208 | M | - | c.3221_3222delAA, p.Lys1074Argfs*27 | - | - | >3^rd^ |  |
| 209 | M | - | c.5957_5958delGA, p.Arg1986llefs*45 | - | - | <3^rd^ |  |
| 210 | M | - | c.3974delT, p.Phe1325Serfs*20 | - | - | <3^rd^ |  |
| 211 | F | - | c.3222dupA, p.Glu1075Argfs*27 | - | - | <3^rd^ |  |
| 212 | M | - | c.2866G>T, p.Glu956* | - | - | <3^rd^ |  |
| 213 | M | - | c.7534C>T, p.Arg2512Trp | - | - | <3^rd^ |  |
| 214 | F | - | c.2650delG, p.Asp884Thrfs*93 | - | - | <3^rd^ |  |
| 215 | F | - | c.3339G>A, p.Trp1113* | - | - | <3^rd^ |  |
| 216 | M | - | c.6071_6084del, p.Pro2024Argfs*3 | - | - | - | [56] |
| 217 | F | - | c.3770_3771delAA, p.Lys1257fs*25 | - | - | - |  |
| 218 | M | - | c.5205delC, p.Val1736Cysfs*227 | - | - | - |  |
| 219 | M | - | c.1903_1907delAAACA, p.Lys635Glnfs*26 | - | - | - |  |
| 220 | F | - | c.1385_1388delCAAA, p.Thr462Lysfs*47 | - | - | - |  |
| 221 | F | - | c.2412delA, p.Glu805Lysfs*58 | - | - | - |  |
| 222 | M | - | c.2175_2178delCAAA, p.Asn725Lysfs*23 | - | - | - |  |
| 223 | M | - | c.2398_2401delGAAA, p.Glu800Asnfs*62 | - | - | - |  |
| 224 | F | - | c.4389_4390delGA, p.Lys1464Thrfs*89 | - | - | - |  |
| 225 | F | - | c.1903_1907delAAACA, p.Lys635Glnfs*26 | - | - | - |  |
| 226 | F | - | c.2175_2178delCAAA, p.Asn725Lysfs*23 | - | - | - |  |
| 227 | F | - | c.1285_1286delTC, p.Ser429Glyfs*8 | - | - | - |  |
| 228 | M | - | c.7416C>G, p.Tyr2472* | - | - | - |  |
| 229 | M | - | c.1977C>G, p.Tyr659* | - | - | - |  |
| 230 | M | - | c.7192C>T, p.Gln2398* | - | - | - |  |
| 231 | F | - | c.3019C>T, p.Arg1007* | - | - | - |  |
| 232 | F | 7 | c.4528_4529delCC, p.P1510Afs*43 | <3^rd^ | 50^th^ | 3^rd^ | [57] |
| 233 | M | 5 | c.3836del, p.Ser1279fs | -0.6 | -1.5 | -2.4 | [58] |
| 234 | M | 7 | c.1903_1907del, p.Lys635Glnfs*26 | 1 | -0.5 | -2.8 |  |
| 235 | M | 7 | c.1903_1907del, p.(Lys635Glnfs*26 | -3 | -2 | -1.5 | [59] |
| 236 | F | 6 | c.2512C>T, p.Arg838* | -0.52 | -0.55 | - | [60] |
| 237 | F | 6 | c.4039_4041del, p.Lys1347del | - | - | -2 | [61] |
| 238 | F | - | c.4039_4041del, p.Lys1347del | - | - | -1.5 |  |
| 239 | F | 9 | c.6427C>G, p. Leu2143Val | -2 | -0.2 | -2 |  |
| 240 | F | - | c.6427C>G, p. Leu2143Val | - | - | -0.8 |  |
| 241 | M | 5 | c.5889del, p.Ile1963Metfs*9 | -1.76 | - | -3.04 | [62] |
| 242 | M | 11 | c3310dup, p.Glu110Glyfs*5 | - | - | 0.77 |  |
| 243 | F | 12 | 16:89351565, p.T461R | - | - | - | [63] |
| 244 | M | 6 | c.2615_2616del, p.S872Cfs*43 | - | - | - | [64] |
| 245 | - | 0.3 | c.1903_1907del, p.Lys635Glnfs*26 | - | - | -2.14 | [65] |
| 246 | - | 2 | c.1903_1907del, p.Lys635Glnfs*26 | - | - | -0.52 |  |
| 247 | - | 0.3 | c.7607G>A, p.Arg2538Gln | - | - | -1.66 |  |
| 248 | - | 7 | c.4558del, p.Asp1520Thrfs*11 | - | - | -3.61 |  |
| 249 | - | 4 | c.2395A>T, p.Lys799* | - | - | -0.48 |  |
| 250 | - | 4 | c.1389dup, p.Gly464Argfs*29 | - | - | <-2 |  |
| 251 | - | 10 | c. 7552C>T, p.Gln2518* | - | - | -2.5 |  |
| 252 | - | 16 | c.2828_2829del, p.Glu943Valfs*74 | - | - | -1.59 |  |
| 253 | - | 8 | c.6340C>T, p.Gln2114* | - | - | >-2 |  |
| 254 | - | 26 | c.3295_3296del, p.Phe1099Leufs*2 | - | - | >-2 |  |
| 255 | - | 8 | c.3771dup, p.Glu1258Argfs*25 | - | - | <-2 |  |
| 256 | - | 22 | c.1385_1388del, p.Thr462Lysfs*47 | - | - | >-2 |  |
| 257 | - | 3 | c.6053_6057del, p.Pro2018Argfs*12 | - | - | - |  |
| 258 | - | - | c.1200G>C, p.Lys400Asn | - | - | - | [66] |
| 259 | - | - | c.2075A>G, p.Asp692Gly | - | - | - |  |
| 260 | - | - | c.3277G>A, p.Gly1093Arg | - | - | - |  |
| 261 | - | - | c.6049G>A, p.Ala2017Thr | - | - | - |  |
| 262 | - | - | c.7388C>A, p.Pro2463His | - | - | - |  |
| 263 | - | - | c.7535G>A, p.Arg2512Gln | - | - | - |  |
| 264 | - | - | c.7535G>A, p.Arg2512Gln | - | - | - |  |
| 265 | - | - | c.7535G>T, p.Arg2512Leu | - | - | - |  |
| 266 | - | - | c.7564G>A, p.Glu2522Lys | - | - | - |  |
| 267 | - | - | c.7564G>A, p.Glu2522Lys | - | - | - |  |
| 268 | - | - | c.7567C>T, p.Arg2523Trp | - | - | - |  |
| 269 | - | - | c.7606C>T, p.Arg2536Trp | - | - | - |  |
| 270 | - | - | c.7607G>C, p.Arg2536Pro | - | - | - |  |
| 271 | - | - | c.7735C>T, p.Arg2579Cys | - | - | - |  |
| 272 | - | - | c.7736G>A, p.Arg2579His | - | - | - |  |
| 273 | - | - | c.7736G>A, p.Arg2579His | - | - | - |  |
| 274 | - | - | c.7736G>A, p.Arg2579His | - | - | - |  |
| 275 | - | - | c.7736G>A, p.Arg2579His | - | - | - |  |
| 276 | - | - | c.7736G>A, p.Arg2579His | - | - | - |  |
| 277 | - | - | c.7736G>A, p.Arg2579His | - | - | - |  |
| 278 | - | - | c.7741C>T, p.Arg2581Cys | - | - | - |  |
| 279 | - | - | c.7753C>T, p.Arg2585Cys | - | - | - |  |
| 280 | - | - | c.7753C>T, p.Arg2585Cys | - | - | - |  |
| 281 | - | - | c.7814T>G, p.Leu2605Arg | - | - | - |  |
| 282 | - | - | c.7814T>G, p.Leu2605Arg | - | - | - |  |
| 283 | M | 23 months | C.1903_1907del, p.Lys635Glnfs*26 | 10-25^th^ | - | 50-75^th^ | [67] |
| 284 | M | 14 months | c.5350_5351delTC, p.Ser1784Hisfs*12 | >90^th^ | - | 75-90^th^ |  |
| 285 | M | 9 | c.6982dup, p.Arg2328Profs*204 | - | - | <-2 | [68] |
| 286 | M | 4 | c.3140_3143del, p.Gln1047Argfs*270 | - | - | -2.38 |  |
| 287 | M | 17 | c.7569+1G>C | - | - | <-2 |  |
| 288 | M | 8 | c.7237C>T, p.Gln2413* | - | - | <-2 |  |
| 289 | F | 13 | c.211_226+1del | - | - | - | [69] |
| 290 | M | 7 | c. 4765C>T, p.Q1589* | - | - | - | [70] |
| 291 | - | - | c.1903_1907delAAACA, p.Lys635Glnfs*26 | - | - | <3^rd^ | [71] |
| 292 | - | - | c.7909_7915dup, p.Leu2639Glnfs*113 | -1.23 | - | -2.39 | [72] |
| 293 | - | - | c.2397del, p.Glu800Lysfs*63 | -0.08 | - | -1.87 |  |
| 294 | - | - | c.2119del, p.Glu707Lysfs*12 | -0.54 | - | -0.76 |  |
| 295 | - | - | c.3170_3171del, p.Lys1057Argfs*10 | -1.29 | - | -2.92 |  |
| 296 | - | - | c.1908_1909del, p.His636Glnfs*26 | -1.66 | - | -2.06 |  |
| 297 | - | - | c.6670del, p.Glu2224Argfs*113 | 0.32 | - | -2.37 |  |
| 298 | - | - | c.4087C>T, p.Arg1363* | 0.16 | - | -4.02 |  |
| 299 | - | - | c.4384dup, p.Arg1462Lysfs*92 | - | - | - | [73] |
| 300 | M | 10 | c.3770_3771del, p.Lys1257Argfs*25 | -2.56 | -2.08 | -2.18 | [74] |
| 301 | - | - | c.5238_5239delGC, p.Pro1747Argfs*49 | - | - | - | [75] |
| 302 | M | 2 | c.316C>T, p.R106* | - | - | <-2 | [76] |
| 303 | - | - | c.1903_1907delAAACA, p.Lys635Glnfs*26 | - | - | - | [77] |
| 304 | - | - | c.4911delT, p.P1638Lfs*48 | - | - | - |  |
| 305 | M | - | c.4398_4401del, p.Glu1467Asnfs*63 | - | - | - | [78] |
| 306 | F | - | c.4398_4401del, p.Glu1467Asnfs*63 | - | - | - |  |
| 307 | F | - | c.4398_4401del, p.Glu1467Asnfs*63 | - | - | - |  |
| 308 | M | - | c.1770_1776del, p.Pro591Gly*60 | - | - | - | [79] |
| 309 | M | 10 | c.2523G > A, p.Trp841* | - | - | >10th | [80] |
| 310 | F | 6 | c.6552_6558dupTGAGGAG, p.Pro2187* | - | - | >10th |  |
| 311 | M | 13 | c.3319_3322delAAAG, p.Lys1107Alafs*210 | - | - | <10th |  |
| 312 | F | 16 | c.4261G > T, p.Glu1421* | - | - | >10th |  |
| 313 | F | 16 | c.6836_6837delTG, p.Val2279Glyfs*16 | - | - | <10th |  |
| 314 | F | 6 | c.1388_1389delAA, p.Lys463Argfs*29 | - | - | <10th |  |
| 315 | M | 8 | c.1903_1907delAAACA, p.Lys635Glnfs*26 | - | - | >10th |  |
| 316 | M | 1 | c.2408_2412delAAAAA, p.Lys803Argfs*5 | - | - | <10^th^ |  |
| 317 | F | 16 | c.2197C > T, p.Arg733* | - | - | >10th |  |
| 318 | M | 11 | c.1459G > T, p.Glu487* | - | - | <10^th^ |  |
| 319 | F | 12 | c.1381_1384delGAAA, p.Glu461Glnfs*48 | - | - | >10th |  |
| 320 | F | 12 | c.1381_1384delGAAA, p.Glu461Glnfs*48 | - | - | >10th |  |
| 321 | M | 16 | c.3768_3769delCA, p.His1256Glnfs*26 | - | - | >10th |  |
| 322 | F | 27 | c.2398_2401delGAAA, p.Glu800Asnfs*62 | - | - | >10^th^ |  |
| 323 | M | 30 | c.1372C > T, p.Arg458* | - | - | <10^th^ |  |
| 324 | F | 14 | c.2368G > T, p.Glu790* | - | - | <10^th^ |  |
| 325 | M | 5 | c.1232C > A, p.Ser411* | - | - | >10^th^ |  |
| 326 | F | 18 | c.7606C > T, p.Arg2536Trp | - | - | <10^th^ |  |
| 327 | F | 16 | c.2280_2281delGT, p.Y761Qfs*20 | <3^rd^ | -4.5 | -3.42 | [81] |
| 328 | F | 39 | c.2280_2281delGT, p.Y761Qfs*20 | - | - | -0.01 |  |
| 329 | M | 57 | c.4093C>T, p.Arg1365* | - | - | - | [82] |
| 330 | F | 7 | c.4964_4965del, p.K1655Rfs*12 | - | - | -2.19 | [83] |
| 331 | F | 1 | c.1757_1776del, p.Val586Glufs*41 | - | - | -1.88 |  |
| 332 | M | 9 | c.3770_3771del, p.Lys1257Argfs*25 | - | - | - | [84] |
| 333 | F | 13 | c.2329_2332del, p.Glu777Argfs*5 | - | - | - |  |
| 334 | M | 8 | c.2344_2345dup, p.Leu782Phefs*2 | - | - | - |  |
| 335 | F | 18 | c.2175_2178del, p.Asn725Lysfs*23 | - | - | - |  |
| 336 | M | 18 | c.3309del, p.Asp1104Metfs*214 | - | - | - |  |
| 337 | F | 14 | c.1920_1921insT, p.Lys641* | - | - | - |  |
| 338 | F | 9 | c.1930_1907del, p.Lys635Glnfs*26 | - | - | - |  |
| 339 | M | 10 | c.3786_3789del, p.Lys1262Asnfs*55 | - | - | - |  |
| 340 | F | 13 | c.3201dupA, p.Asn1068Argfs*34 | - | - | - |  |
| 341 | F | 11 | c.1903_1907del, p.Lys635Glnfs*26 | - | - | - |  |
| 342 | F | 4 | c.1902_1905del, p.Lys635Thrfs*17 | - | - | - |  |
| 343 | F | 2 | c.2398_2401del, p.Glu800Asnfs*62 | - | - | - |  |
| 344 | M | 15.5 | c.4374delG, p.Lys1459Argfs*72 | - | - | - |  |
| 345 | M | 3.4 | c.5174dupC, p.Ser1726Valfs*6 | - | - | - |  |
| 346 | M | 43 | c.1902_1905del, p.Lys635Thrfs*17 | - | - | - |  |
| 347 | F | 41 | c.3786_3789del, p.Lys1262Asnfs*55 | - | - | - |  |
| 348 | F | 16 | c.226+1G>T | - | - | - |  |
| 349 | M | 1 | c.3860_3861delAG, p.Glu1287Glyfs*4 | - | - | - |  |
| 350 | M | 13 | c.6738delA, p.Glu2247Asnfs*90 | - | - | - |  |
| 351 | M | 17 | c.2178del, p.Asp727Thrfs*22 | - | - | - |  |
| 352 | F | 14 | c.1903_1907del, p.Lys635Glnfs*26 | - | - | - |  |
| 353 | - | 12 | c.2404_2407del, p.Leu802Lysfs*60 | 37^th^ | 13^th^ | - | [85] |
| 354 | F | 1 month | c.1896_1897delTA, p.H632Qfs*30 | 1400 g | - | - | [86] |
| 355 | - | - | p.E169* | - | - | - | [87] |
| 356 | - | - | p.S429Gfs*8 | - | - | - |  |
| 357 | - | - | p.E461Qfs*48 | - | - | - |  |
| 358 | - | - | p.S486* | - | - | - |  |
| 359 | - | - | p.E487Vfs*22 | - | - | - |  |
| 360 | - | - | p.S528Rfs*51 | - | - | - |  |
| 361 | - | - | p.K635Qfs*26 | - | - | - |  |
| 362 | - | - | p.Y659* | - | - | - |  |
| 363 | - | - | p.E698Tfs*2 | - | - | - |  |
| 364 | - | - | p.K789Rfs*7 | - | - | - |  |
| 365 | - | - | p.K803Rfs*5 | - | - | - |  |
| 366 | - | - | p.E805Kfs*58 | - | - | - |  |
| 367 | - | - | p.K1009Rfs*310 | - | - | - |  |
| 368 | - | - | p.R1019Gfs*14 | - | - | - |  |
| 369 | - | - | p.D1104Rfs*2 | - | - | - |  |
| 370 | - | - | p.K1170Sfs*147 | - | - | - |  |
| 371 | - | - | p.R1188* | - | - | - |  |
| 372 | - | - | p.D1231Efs*3 | - | - | - |  |
| 373 | - | - | p.S1260* | - | - | - |  |
| 374 | - | - | p.E1263Tfs*19 | - | - | - |  |
| 375 | - | - | p.S1279Kfs*4 | - | - | - |  |
| 376 | - | - | p.K1373* | - | - | - |  |
| 377 | - | - | p.Y1402* | - | - | - |  |
| 378 | - | - | p.K1464fs*89 | - | - | - |  |
| 379 | - | - | p.P1510Afs*43 | - | - | - |  |
| 380 | - | - | p.K1655Rfs*12 | - | - | - |  |
| 381 | - | - | p.P1938Qfs*10 | - | - | - |  |
| 382 | - | - | p.A2265Rfs*8 | - | - | - |  |
| 383 | - | - | p.V2279Gfs*16 | - | - | - |  |
| 384 | - | - | p.R2328* | - | - | - |  |
| 385 | - | - | p.Q2350* | - | - | - |  |
| 386 | - | - | p.Q2397* | - | - | - |  |
| 387 | - | - | p.Q2398* | - | - | - |  |
| 388 | - | - | p.Q2406* | - | - | - |  |
| 389 | - | - | p.P2495Sfs*37 | - | - | - |  |
| 390 | - | - | p.R2512Q | - | - | - |  |
| 391 | - | - | p.R2536W | - | - | - |  |
| 392 | - | - | p.V2649M | - | - | - |  |
| 393 | M | 4 | p.Lys635fs | - | - | - | [88] |
| 394 | M | 9 | p.Lys1257Argfs*25 | - | - | - |  |
| 395 | F | 3 | p.Arg440* | - | - | - |  |
| 396 | F | 6 | p.Glu777Argfs*5 | - | - | - |  |
| 397 | M | 15 | p.E2158* | - | - | - |  |
| 398 | M | 6 | p.Arg2452Gly | - | - | - |  |
| 399 | F | 31 | p.Ser1745Hisfs*51 | - | - | - |  |
| 400 | F | 61 | p.Ser1745Hisfs*51 | - | - | - |  |
| 401 | F | 5 | p.Leu802Lysfs*60 | - | - | - |  |
| 402 | M | 7 | p.Glu2210* | - | - | - |  |
| 403 | F | 3 | p.Asp1331Thrfs*14 | - | - | - |  |
| 404 | F | 2 | p.Asn394llefs*33 | - | - | - |  |
| 405 | F | 21 | p.Lys726Argfs*15 | - | - | - |  |
| 406 | M | 21 | p.Thr340Asnfs*9 | - | - | - |  |
| 407 | F | 8 | p.Lys635Glnfs*26 | - | - | - |  |
| 408 | M | 11 | p.Gln2609* | - | - | - |  |
| 409 | M | 5 | p.Arg1497Glyfs*56 | - | - | - |  |
| 410 | M | 6 | p.Arg440* | - | - | - |  |
| 411 | M | 12 | p.Gly2006Argfs*26 | - | - | - |  |
| 412 | F | 5 | p.Glu0805Argfs*57 | - | - | - |  |
| 413 | M | 5 | p.Lys1257Argfs*25 | - | - | - |  |
| 414 | M | 5 | p.Arg2536Gln | - | - | - |  |
| 415 | M | 24 | p.Arg1462Lysfs*92 | - | - | - |  |
| 416 | F | 6 | p.Glu800Asnfs*62 | - | - | - |  |
| 417 | M | 4 | p.Asn725Lysfs*23 | - | - | - |  |
| 418 | M | 21 | p.Lys2597* | - | - | - |  |
| 419 | M | 55 | p.Lys2597* | - | - | - |  |
| 420 | F | 11 | p.Arg759Glu*23fs | - | - | - |  |
| 421 | F | 20 | p.Ala2201Cysfs*6 | - | - | - |  |
| 422 | F | 11 | p.Gln1887* | - | - | - |  |
| 423 | M | 5 | p.Gln1743* | - | - | - |  |
| 424 | M | 16 | p.Glu1075Glyfs*242 | - | - | - |  |
| 425 | F | 5 | p.Tyr659* | - | - | - |  |
| 426 | M | 5 | p.Glu805Argfs*57 | - | - | - |  |
| 427 | M | 20 | p.Lys1257Argfs*25 | - | - | - |  |
| 428 | M | 3 | p.Arg1466Glyfs*87 | - | - | - |  |
| 429 | F | 16 | p.Lys631fs | - | - | - |  |
| 430 | F | 30 | p.Gln1743* | - | - | - |  |
| 431 | M | 18 | c.2398_2410del, p.Glu800Asnfs*62 | - | - | - | [89] |
| 432 | M | 6 | c.3860_3861delAG, p.Glu1287Glyfs*4 | - | - | - |  |
| 433 | F | 10 | c.6836_6837delTG, p.Val2279GlyfsTer16 | - | - | - |  |
| 434 | M | 6.5 | c.6917_6942del26, p.P2306Rfs*217 | - | - | - |  |
| 435 | M | 22 months | c.4640delA, p.H1487Lfs*44 | - | - | - | [90] |
| 436 | F | 8 | c.7799G>A, p.R2600H | - | - | <3rd |  |
| 437 | M | 15 | c.7192C>T, p.Gln2398* | - | - | <3rd |  |
| 438 | F | 7 | c.1903_1907delAAACA, p.Lys635Glnfs*26 | - | - | >3rd |  |
| 439 | M | 4 | c.4498C>T, p.Gln1500* | - | - | >3rd |  |
| 440 | F | 1 | c.4107_4108delGA, p.K1370Gfs*15 | - | - | >3rd |  |
| 441 | M | 4 | c.6071_6084del, p.P2024Rfs*3 | - | - | >3rd |  |
| 442 | M | 13 | c.2398_2401delGAAA, p.Glu800Asnfs*62 | - | - | <3rd |  |
| 443 | M | 1 | c.706_707dupAC, p.P237Rfs*15 | - | - | <3rd |  |
| 444 | M | 2 | c.7874_7877delAGCT, p.Q2625Rfs*16 | - | - | >3rd |  |
| 445 | M | 12 | c.6792delC, p.Ala2265Profs*72 | - | - | - | [91] |
| 446 | M | - | c.6968_6975delCCCCGAAG, p.Ala2323fs | - | - | - | [92] |
| 447 | M | 8 | c.1669C>G, p.Pro557Ala | - | - | - | [93] |
| 448 | F | 11 | c.3907del, p.Val1303Serfs*15 | - | - | <3rd |  |
| 449 | F | 2 | c.397+1G>A | - | -1.42 | -1.79 | [94] |
| 450 | M | 10 | c.226+1G>A | - | - | -0.88 |  |
| 451 | F | 9 | c.2454dup, p.Asn819fs*1 | - | -0.62 | 0.65 |  |
| 452 | F | 7 | c.2647del, p.Glu883Argfs*94 | - | -2.88 | -2.37 |  |
| 453 | F | 37 | c.2647del, p.Glu883Argfs*94 | - | - | -2.29 |  |
| 454 | F | 3 months | c.4093C>T, p.Arg1365* | - | -0.66 | -1.49 |  |
| 455 | M | - | c.7595A>C, p.Gln2532Pro | - | - | - | [95] |
| 456 | - | - | c.6270delT, p.Q209Rfs*84 | - | - | - | [96] |
| 457 | M | - | c.6858delC, p.D2286Efs*51 | - | - | - |  |
| ***ANKRD11* variants in ClinVar database** | | | | | | | |
| 1 |  |  | c.7944C>A, p.Tyr2648* |  |  |  |  |
| 2 |  |  | c.7868_7872del, p.Glu2623fs |  |  |  |  |
| 3 |  |  | c.7812C>A, p.Cys2604* |  |  |  |  |
| 4 |  |  | c.7806+1G>T |  |  |  |  |
| 5 |  |  | c.7789A>T, p.Lys2597* |  |  |  |  |
| 6 |  |  | c.7788_7804del, p.Asp2596fs |  |  |  |  |
| 7 |  |  | c.7765T>C, p.Ser2589Pro |  |  |  |  |
| 8 |  |  | c.7751C>A, p.Ala2584Asp |  |  |  |  |
| 9 |  |  | c.7714-2A>G |  |  |  |  |
| 10 |  |  | c.7714-1G>C |  |  |  |  |
| 11 |  |  | c.7713+2T>C |  |  |  |  |
| 12 |  |  | c.7711C>T, p.Gln2571* |  |  |  |  |
| 13 |  |  | c.7684G>A, p.Glu2562Lys |  |  |  |  |
| 14 |  |  | c.7636dup, p.Ala2546fs |  |  |  |  |
| 15 |  |  | c.7617_7629del, p.His2538_Cys2539ins* |  |  |  |  |
| 16 |  |  | c.7570-1G>C |  |  |  |  |
| 17 |  |  | c.7570-1G>A |  |  |  |  |
| 18 |  |  | c.7569+1G>A |  |  |  |  |
| 19 |  |  | c.7556A>T, p.His2519Leu |  |  |  |  |
| 20 |  |  | c.7545delG, p.R2516Vfs*11 |  |  |  |  |
| 21 |  |  | c.7544T>C, p.Leu2515Pro |  |  |  |  |
| 22 |  |  | c.7523A>T, p.Gln2508Leu |  |  |  |  |
| 23 |  |  | c.7519C>T, p.Gln2507Ter |  |  |  |  |
| 24 |  |  | c.7480C>A, p.Pro2494Thr |  |  |  |  |
| 25 |  |  | c.7481C>G, p.Pro2494Arg |  |  |  |  |
| 26 |  |  | c.7471-2A>G |  |  |  |  |
| 27 |  |  | c.7470+1G>C |  |  |  |  |
| 28 |  |  | c.7470+5G>C |  |  |  |  |
| 29 |  |  | C.7470+2T>C |  |  |  |  |
| 30 |  |  | c.7433T>C, p.Leu2478Pro |  |  |  |  |
| 31 |  |  | c.7407C>G, p.Tyr2469* |  |  |  |  |
| 32 |  |  | c.7388C>G, p.Pro2463Arg |  |  |  |  |
| 33 |  |  | c.7355G>C, p.Arg2452Pro |  |  |  |  |
| 34 |  |  | c.7354C>T, p.Arg2452Cys |  |  |  |  |
| 35 |  |  | c.7327C>T, p.Gln2443* |  |  |  |  |
| 36 |  |  | c.7311_7314dup, p.Phe2439fs |  |  |  |  |
| 37 |  |  | c.7303del, p.Ala2435fs |  |  |  |  |
| 38 |  |  | c.7290C>G, p.Tyr2430* |  |  |  |  |
| 39 |  |  | c.7267A>T, p.Lys2423* |  |  |  |  |
| 40 |  |  | c.7244_7247del, p.Leu2415fs |  |  |  |  |
| 41 |  |  | c.7234C>T, p.Gln2412* |  |  |  |  |
| 42 |  |  | c.7225G>T, p.Glu2409* |  |  |  |  |
| 43 |  |  | c.7222_7226delinsGGGG, p.Arg2408fs |  |  |  |  |
| 44 |  |  | c.7183C>T, p.Gln2395* |  |  |  |  |
| 45 |  |  | c.7180C>T, p.Gln2394* |  |  |  |  |
| 46 |  |  | c.7168C>T, p.Gln2390* |  |  |  |  |
| 47 |  |  | c.7144dup, p.Gln2382fs |  |  |  |  |
| 48 |  |  | c.7144C>T, p.Gln2382* |  |  |  |  |
| 49 |  |  | c.7138C>T, p.Gln2380* |  |  |  |  |
| 50 |  |  | c.7114dup, p.Arg2372fs |  |  |  |  |
| 51 |  |  | c.7103_7106del, p.Arg2368fs |  |  |  |  |
| 52 |  |  | c.7083dup, p.Thr2362fs |  |  |  |  |
| 53 |  |  | c.7062dup, p.Ser2355fs |  |  |  |  |
| 54 |  |  | c.7062del, p.Ser2355fs |  |  |  |  |
| 55 |  |  | c.7048_7050del, p.Gln2350del |  |  |  |  |
| 56 |  |  | c.6968_6975dup, p.Ala2326fs |  |  |  |  |
| 57 |  |  | c.6928_6934del, p.Ile2310fs |  |  |  |  |
| 58 |  |  | c.6923del, p.Gly2308fs |  |  |  |  |
| 59 |  |  | c.6911_6936del, p.Glu2304fs |  |  |  |  |
| 60 |  |  | c.6882_6883del, p.Glu2295fs |  |  |  |  |
| 61 |  |  | c.6871G>T, p.Glu2291* |  |  |  |  |
| 62 |  |  | c.6847C>T, p.Gln2283* |  |  |  |  |
| 63 |  |  | c.6833_6834del, p.Thr2278fs |  |  |  |  |
| 64 |  |  | c.6807_6808del, p.Ala2270fs |  |  |  |  |
| 65 |  |  | c.6793del, p.Ala2265fs |  |  |  |  |
| 66 |  |  | c.6786_6787insA, p.Pro2263fs |  |  |  |  |
| 67 |  |  | c.6772dup, p.Ala2258fs |  |  |  |  |
| 68 |  |  | c.6760G>T, p.Gly2254* |  |  |  |  |
| 69 |  |  | c.6748_6757del, p.Pro2250fs |  |  |  |  |
| 70 |  |  | c.6742C>T, p.Gln2248* |  |  |  |  |
| 71 |  |  | c.6739_6740insCC, p.Glu2247fs |  |  |  |  |
| 72 |  |  | c.6688_6689del, p.Arg2230fs |  |  |  |  |
| 73 |  |  | c.6685G>T, p.Glu2229* |  |  |  |  |
| 74 |  |  | c.6677_6680delinsAGA, p.Val2226fs |  |  |  |  |
| 75 |  |  | c.6670G>T, p.Glu2224* |  |  |  |  |
| 76 |  |  | c.6624_6625del, p.Glu2210fs |  |  |  |  |
| 77 |  |  | c.6623C>A, p.Ser2208* |  |  |  |  |
| 78 |  |  | c.6569_6609dup, p.Glu2204fs |  |  |  |  |
| 79 |  |  | c.6596_6597insA, p.Ala2201fs |  |  |  |  |
| 80 |  |  | c.6589dup, p.Thr2197fs |  |  |  |  |
| 81 |  |  | c.6580C>Tp.Gln2194* |  |  |  |  |
| 82 |  |  | c.6513del, p.Val2173fs |  |  |  |  |
| 83 |  |  | c.6469del, p.Glu2157fs |  |  |  |  |
| 84 |  |  | c.6465del, p.Val2156fs |  |  |  |  |
| 85 |  |  | c.6436A>C, p.Lys2146Gln |  |  |  |  |
| 86 |  |  | c.6430C>T, p.Gln2144* |  |  |  |  |
| 87 |  |  | c.6401_6422dup, p.Pro2142fs |  |  |  |  |
| 88 |  |  | c.6402del, p.Phe2136fs |  |  |  |  |
| 89 |  |  | c.6349_6362del, p.Pro2117fs |  |  |  |  |
| 90 |  |  | c.6298_6299dup, p.Asn2100fs |  |  |  |  |
| 91 |  |  | c.6320dup, p.Gly2108fs |  |  |  |  |
| 92 |  |  | c.6288_6289insT, p.Pro2097fs |  |  |  |  |
| 93 |  |  | c.6249del, p.Ala2084fs |  |  |  |  |
| 94 |  |  | c.6218del, p.Pro2073fs |  |  |  |  |
| 95 |  |  | c.6212C>G, p.Ser2071* |  |  |  |  |
| 96 |  |  | c.6210_6211del, p.Lys2070fs |  |  |  |  |
| 97 |  |  | c.6197_6198delinsAA, p.Phe2066* |  |  |  |  |
| 98 |  |  | c.6177del, p.Ser2060fs |  |  |  |  |
| 99 |  |  | c.6165del, p.Tyr2056fs |  |  |  |  |
| 100 |  |  | c.6152dup, p.Glu2052fs |  |  |  |  |
| 101 |  |  | c.6138del, p.Ala2047fs |  |  |  |  |
| 102 |  |  | c.6101del, p.Leu2034fs |  |  |  |  |
| 103 |  |  | c.6067_6081delinsCC, p.Ala2023fs |  |  |  |  |
| 104 |  |  | c.6045C>A, p.Tyr2015* |  |  |  |  |
| 105 |  |  | c.6031_6041del, p.Ser2011fs |  |  |  |  |
| 106 |  |  | c.5974A>T, p.Lys1992* |  |  |  |  |
| 107 |  |  | c.5974A>C, p.Lys1992Gln |  |  |  |  |
| 108 |  |  | c.5865del, p.Glu1955fs |  |  |  |  |
| 109 |  |  | c.5859del, p.Ser1954fs |  |  |  |  |
| 110 |  |  | c.5833G>T, p.Glu1945* |  |  |  |  |
| 111 |  |  | c.5813_5817del, p.Pro1938fs |  |  |  |  |
| 112 |  |  | c.5790C>G, p.Tyr1930* |  |  |  |  |
| 113 |  |  | c.5777dup, p.Glu1927fs |  |  |  |  |
| 114 |  |  | c.5739_5766del, p.Glu1915fs |  |  |  |  |
| 115 |  |  | c.5682dup, p.Arg1895fs |  |  |  |  |
| 116 |  |  | c.5667del, p.Lys1889fs |  |  |  |  |
| 117 |  |  | c.5651C>G, p.Ser1884* |  |  |  |  |
| 118 |  |  | c.5647_5651del, p.Phe1883fs |  |  |  |  |
| 119 |  |  | c.5637dup, p.Glu1880fs |  |  |  |  |
| 120 |  |  | c.5564_5565del, p.Tyr1855fs |  |  |  |  |
| 121 |  |  | c.5560_5563del, p.Asp1854fs |  |  |  |  |
| 122 |  |  | c.5552_5556del, p.Tyr1851fs |  |  |  |  |
| 123 |  |  | c.5550C>G, p.Tyr1850* |  |  |  |  |
| 124 |  |  | c.5537del, p.Leu1846fs |  |  |  |  |
| 125 |  |  | c.5524A>T, p.Lys1842* |  |  |  |  |
| 126 |  |  | c.5504del, p.Leu1835fs |  |  |  |  |
| 127 |  |  | c.5494_5495del, p.Arg1832fs |  |  |  |  |
| 128 |  |  | c.5469del, p.Met1825fs |  |  |  |  |
| 129 |  |  | c.5469dup, p.Pro1824fs |  |  |  |  |
| 130 |  |  | c.5447del, p.Pro1816fs |  |  |  |  |
| 131 |  |  | c.5437C>T, p.Gln1813* |  |  |  |  |
| 132 |  |  | c.5438_5439insT, p.Gln1813fs |  |  |  |  |
| 133 |  |  | c.5375_5376del, p.Ser1792fs |  |  |  |  |
| 134 |  |  | c.5364C>G, p.Tyr1788* |  |  |  |  |
| 135 |  |  | c.5334_5344del, p.Pro1779fs |  |  |  |  |
| 136 |  |  | c.5317G>T, p.Glu1773* |  |  |  |  |
| 137 |  |  | c.5303_5313del, p.Ser1768fs |  |  |  |  |
| 138 |  |  | c.5233_5234del, p.Ser1745fs |  |  |  |  |
| 139 |  |  | c.5227C>T, p.Gln1743* |  |  |  |  |
| 140 |  |  | c.5174dup, p.Ser1726fs |  |  |  |  |
| 141 |  |  | c.5117dup, p.Thr1707fs |  |  |  |  |
| 142 |  |  | c.5117del, p.Pro1706fs |  |  |  |  |
| 143 |  |  | c.5105dup, p.Val1703fs |  |  |  |  |
| 144 |  |  | c.5089C>T, p.Gln1697* |  |  |  |  |
| 145 |  |  | c.5065del, p.Leu1689fs |  |  |  |  |
| 146 |  |  | c.5053del, p.His1684_Met1685ins* |  |  |  |  |
| 147 |  |  | c.5030_5031del, p.Lys1677fs |  |  |  |  |
| 148 |  |  | c.4947_4948dup, p.Lys1650fs |  |  |  |  |
| 149 |  |  | c.4933del, p.Leu1645fs |  |  |  |  |
| 150 |  |  | c.4902del, p.Leu1635fs |  |  |  |  |
| 151 |  |  | c.4875del, p.Lys1625fs |  |  |  |  |
| 152 |  |  | c.4786G>T, p.Glu1596* |  |  |  |  |
| 153 |  |  | c.4750G>T, p.Glu1584* |  |  |  |  |
| 154 |  |  | c.4742_4743insAT, p.Phe1583fs |  |  |  |  |
| 155 |  |  | c.4634dup, p.Val1546fs |  |  |  |  |
| 156 |  |  | c.4634del, p.Pro1545fs |  |  |  |  |
| 157 |  |  | c.4624_4625del, p.Lys1542fs |  |  |  |  |
| 158 |  |  | c.4621_4624del, p.Glu1541fs |  |  |  |  |
| 159 |  |  | c.4619_4620del, p.Lys1540fs |  |  |  |  |
| 160 |  |  | c.4585A>T, p.Lys1529* |  |  |  |  |
| 161 |  |  | c.4554del, p.Arg1519fs |  |  |  |  |
| 162 |  |  | c.4489_4490del, p.Arg1497fs |  |  |  |  |
| 163 |  |  | c.4396_4397del, p.Arg1466fs |  |  |  |  |
| 164 |  |  | c.4381_4384del, p.Lys1461fs |  |  |  |  |
| 165 |  |  | c.4381_4382del, p.Lys1461fs |  |  |  |  |
| 166 |  |  | c.4372_4375del, p.Glu1458fs |  |  |  |  |
| 167 |  |  | c.4370_4373del, p.Lys1457fs |  |  |  |  |
| 168 |  |  | c.4369A>T, p.Lys1457* |  |  |  |  |
| 169 |  |  | c.4349_4352del, p.Ser1450fs |  |  |  |  |
| 170 |  |  | c.4345_4346del, p.Gly1449fs |  |  |  |  |
| 171 |  |  | c.4334dup, p.Pro1447fs |  |  |  |  |
| 172 |  |  | c.4333_4336del, p.Leu1445fs |  |  |  |  |
| 173 |  |  | c.4306_4307del, p.Glu1436fs |  |  |  |  |
| 174 |  |  | c.4306G>T, p.Glu1436* |  |  |  |  |
| 175 |  |  | c.4288A>T, p.Lys1430* |  |  |  |  |
| 176 |  |  | c.4270del, p.Ser1424fs |  |  |  |  |
| 177 |  |  | c.4205dup, p.Tyr1402* |  |  |  |  |
| 178 |  |  | c.4189del, p.Leu1397fs |  |  |  |  |
| 179 |  |  | c.4176C>G, p.Tyr1392* |  |  |  |  |
| 180 |  |  | c.4145dup, p.Gly1384fs |  |  |  |  |
| 181 |  |  | c.4148_4151del, p.Gly1383fs |  |  |  |  |
| 182 |  |  | c.4140C>G, p.Tyr1380* |  |  |  |  |
| 183 |  |  | c.4109_4124dup, p.Lys1376fs |  |  |  |  |
| 184 |  |  | c.4114G>T, p.Glu1372* |  |  |  |  |
| 185 |  |  | c.4091_4092del, p.Glu1364fs |  |  |  |  |
| 186 |  |  | c.4067del, p.Ser1356fs |  |  |  |  |
| 187 |  |  | c.4059del, p.Ser1354fs |  |  |  |  |
| 188 |  |  | c.4055_4058del, p.His1352fs |  |  |  |  |
| 189 |  |  | c.4052_4053insT, p.Arg1351fs |  |  |  |  |
| 190 |  |  | c.4016dup, p.Cys1339fs |  |  |  |  |
| 191 |  |  | c.3948del, p.Gly1316_Leu1317ins* |  |  |  |  |
| 192 |  |  | c.3933_3945del, p.Gly1312_Gln1313ins* |  |  |  |  |
| 193 |  |  | c.3936dup, p.Gln1313fs |  |  |  |  |
| 194 |  |  | c.3882_3885dup, p.Ser1296fs |  |  |  |  |
| 195 |  |  | c.3843dup, p.Glu1282* |  |  |  |  |
| 196 |  |  | c.3829del, p.Glu1277fs |  |  |  |  |
| 197 |  |  | c.3812C>A, p.Ser1271* |  |  |  |  |
| 198 |  |  | c.3787_3788del, p.Glu1263fs |  |  |  |  |
| 199 |  |  | c.3760_3776del, p.Ser1254fs |  |  |  |  |
| 200 |  |  | c.3770_3773del, p.Lys1257fs |  |  |  |  |
| 201 |  |  | c.3708_3709del, p.Lys1237fs |  |  |  |  |
| 202 |  |  | c.3706C>T, p.Gln1236* |  |  |  |  |
| 203 |  |  | c.3702_3705del, p.Lys1235fs |  |  |  |  |
| 204 |  |  | c.3693_3697del, p.Asp1231fs |  |  |  |  |
| 205 |  |  | c.3651C>A, p.Tyr1217* |  |  |  |  |
| 206 |  |  | c.3603_3604del, p.Val1201_Phe1202ins* |  |  |  |  |
| 207 |  |  | c.3597_3598del, p.Lys1200fs |  |  |  |  |
| 208 |  |  | c.3632_3633del, p.Lys1211fs |  |  |  |  |
| 209 |  |  | c.3603dup, p.Phe1202fs |  |  |  |  |
| 210 |  |  | c.3590_3594del, p.Lys1197fs |  |  |  |  |
| 211 |  |  | c.3586_3587del, p.Asp1196fs |  |  |  |  |
| 212 |  |  | c.3562_3563insA, p.Arg1188fs |  |  |  |  |
| 213 |  |  | c.3562C>T, p.Arg1188* |  |  |  |  |
| 214 |  |  | c.3547_3548del, p.Asp1183fs |  |  |  |  |
| 215 |  |  | c.3532_3535dup, p.Lys1179fs |  |  |  |  |
| 216 |  |  | c.3509_3512del, p.Lys1170fs |  |  |  |  |
| 217 |  |  | c.3425_3431delinsTCCGTCCTG, p.Asp1142fs |  |  |  |  |
| 218 |  |  | c.3369_3372del, p.Ser1123fs |  |  |  |  |
| 219 |  |  | c.3367_3368del, p.Glu1122_Ser1123ins* |  |  |  |  |
| 220 |  |  | c.3334dup, p.Ser1112fs |  |  |  |  |
| 221 |  |  | c.3306_3309del, p.Lys1103fs |  |  |  |  |
| 222 |  |  | c.3309del, p.Asp1104fs |  |  |  |  |
| 223 |  |  | c.3268del, p.Ala1090fs |  |  |  |  |
| 224 |  |  | c.3244C>T, p.Gln1082* |  |  |  |  |
| 225 |  |  | c.3240del, p.Asp1081fs |  |  |  |  |
| 226 |  |  | c.3239_3240del, p.Leu1080fs |  |  |  |  |
| 227 |  |  | c.3228_3232del, p.Arg1076fs |  |  |  |  |
| 228 |  |  | c.3219_3220del, p.Asp1073fs |  |  |  |  |
| 229 |  |  | c.3198_3199del, p.His1066fs |  |  |  |  |
| 230 |  |  | c.3187A>T, p.Lys1063* |  |  |  |  |
| 231 |  |  | c.3151G>T, p.Glu1051* |  |  |  |  |
| 232 |  |  | c.3138T>A, p.Cys1046* |  |  |  |  |
| 233 |  |  | c.3127del, p.Leu1043fs |  |  |  |  |
| 234 |  |  | c.3122C>A, p.Ser1041* |  |  |  |  |
| 235 |  |  | c.3096del, p.Ser1033fs |  |  |  |  |
| 236 |  |  | c.3084C>A, p.Tyr1028* |  |  |  |  |
| 237 |  |  | c.3084del, p.Arg1027_Tyr1028ins* |  |  |  |  |
| 238 |  |  | c.3084C>A, p.Tyr1028* |  |  |  |  |
| 239 |  |  | c.3066del, p.Thr1023fs |  |  |  |  |
| 240 |  |  | c.3055_3059del, p.Arg1019fs |  |  |  |  |
| 241 |  |  | c.3024_3025dup, p.Lys1009fs |  |  |  |  |
| 242 |  |  | c.2944G>T, p.Glu982* |  |  |  |  |
| 243 |  |  | c.2904del, p.Glu969fs |  |  |  |  |
| 244 |  |  | c.2857del, p.Asp953fs |  |  |  |  |
| 245 |  |  | c.2853­_2856del, p.Lys952fs |  |  |  |  |
| 246 |  |  | c.2826_2829del, p.Arg942fs |  |  |  |  |
| 247 |  |  | c.2810del, p.Lys937fs |  |  |  |  |
| 248 |  |  | c.2803dup, p.Ser935fs |  |  |  |  |
| 249 |  |  | c.2793dup, p.Gly932fs |  |  |  |  |
| 250 |  |  | c.2777del, p.Lys926fs |  |  |  |  |
| 251 |  |  | c.2728_2729del, p.Arg910fs |  |  |  |  |
| 252 |  |  | c.2719_2730delinsT, p.Arg906_Lys907ins* |  |  |  |  |
| 253 |  |  | c.2716C>T, p.Arg906* |  |  |  |  |
| 254 |  |  | c.2704G>T, p.Glu902* |  |  |  |  |
| 255 |  |  | c.2662_2666del, p.Arg888fs |  |  |  |  |
| 256 |  |  | c.2647G>T, p.Glu883* |  |  |  |  |
| 257 |  |  | c.2647del, p.Glu883fs |  |  |  |  |
| 258 |  |  | c.2618_2619del, p.Val873fs |  |  |  |  |
| 259 |  |  | c.2609_2612dup, p.Asp871fs |  |  |  |  |
| 260 |  |  | c.2607dup, p.Ser870fs |  |  |  |  |
| 261 |  |  | c.2589dup, p.Asp864fs |  |  |  |  |
| 262 |  |  | c.2563del, p.Glu855fs |  |  |  |  |
| 263 |  |  | c.2553_2556del, p.Asp851fs |  |  |  |  |
| 264 |  |  | c.2536del, p.Ser846fs |  |  |  |  |
| 265 |  |  | c.2518del, p.Arg840fs |  |  |  |  |
| 266 |  |  | c.2516_2517del, p.Asp839fs |  |  |  |  |
| 267 |  |  | c.2500del, p.Ser834fs |  |  |  |  |
| 268 |  |  | c.2494del, p.Ser832fs |  |  |  |  |
| 269 |  |  | c.2464C>T, p.Gln822* |  |  |  |  |
| 270 |  |  | c.2454dup, p.Asn819* |  |  |  |  |
| 271 |  |  | c.2434del, p.Ser812fs |  |  |  |  |
| 272 |  |  | c.2409_2412del, p.Glu805fs |  |  |  |  |
| 273 |  |  | c.2413G>T, p.Glu805* |  |  |  |  |
| 274 |  |  | c.2404_2407del, p.Leu802fs |  |  |  |  |
| 275 |  |  | c.2350G>T, p.Glu784* |  |  |  |  |
| 276 |  |  | c.2329_2332del, p.Glu777fs |  |  |  |  |
| 277 |  |  | c.2329G>T, p.Glu777* |  |  |  |  |
| 278 |  |  | c.2327T>G, p.Leu776* |  |  |  |  |
| 279 |  |  | c.2299A>T, p.Lys767* |  |  |  |  |
| 280 |  |  | c.2297_2300del, p.Lys766fs |  |  |  |  |
| 281 |  |  | c.2288_2289del, p.Glu763fs |  |  |  |  |
| 282 |  |  | c.2283C>G, p.Tyr761* |  |  |  |  |
| 283 |  |  | c.2273dup, p.Arg759fs |  |  |  |  |
| 284 |  |  | c.2273_2274del, p.Leu758fs |  |  |  |  |
| 285 |  |  | c.2262_2265del, p.Glu755fs |  |  |  |  |
| 286 |  |  | c.2216C>A, p.Ser739* |  |  |  |  |
| 287 |  |  | c.2184del, p.Ser729fs |  |  |  |  |
| 288 |  |  | c.2177_2178del, p.Lys726fs |  |  |  |  |
| 289 |  |  | c.2166dup, p.Asp723fs |  |  |  |  |
| 290 |  |  | c.2161dup, p.Ile721fs |  |  |  |  |
| 291 |  |  | c.2130G>A, p.Trp710* |  |  |  |  |
| 292 |  |  | c.2092_2096del, p.Glu698fs |  |  |  |  |
| 293 |  |  | c.2089G>T, p.Glu697* |  |  |  |  |
| 294 |  |  | c.2088dup, p.Glu697fs |  |  |  |  |
| 295 |  |  | c.2073dup, p.Asp692fs |  |  |  |  |
| 296 |  |  | c.2063dup, p.His689fs |  |  |  |  |
| 297 |  |  | c.2054_2055del, p.Lys685fs |  |  |  |  |
| 298 |  |  | c.2047_2048del, p.Lys683fs |  |  |  |  |
| 299 |  |  | c.2022dup, p.Glu675fs |  |  |  |  |
| 300 |  |  | c.2006A>C, p.Asp669Ala |  |  |  |  |
| 301 |  |  | c.1996C>T, p.Gln666* |  |  |  |  |
| 302 |  |  | c.1984del, p.Glu662fs |  |  |  |  |
| 303 |  |  | c.1974_1977dup, p.Glu660fs |  |  |  |  |
| 304 |  |  | c.1976dup, p.Tyr659* |  |  |  |  |
| 305 |  |  | c.1977del, p.Thr658_Tyr659ins* |  |  |  |  |
| 306 |  |  | c.1970_1971del, p.Phe657fs |  |  |  |  |
| 307 |  |  | c.1948C>T, p.Gln650* |  |  |  |  |
| 308 |  |  | c.1901_1904del, p.Thr634fs |  |  |  |  |
| 309 |  |  | c.1896_1897del, p.His632fs |  |  |  |  |
| 310 |  |  | c.1861A>T, p.Lys621* |  |  |  |  |
| 311 |  |  | c.1800dup, p.Arg601fs |  |  |  |  |
| 312 |  |  | c.1742_1743del, p.Ser580_Ser581ins* |  |  |  |  |
| 313 |  |  | c.1731dup, p.Asp578* |  |  |  |  |
| 314 |  |  | c.1726G>T, p.Glu576* |  |  |  |  |
| 315 |  |  | c.1708_1711del, p.Thr571fs |  |  |  |  |
| 316 |  |  | c.1686del, p.Ser563fs |  |  |  |  |
| 317 |  |  | c.1679C>G, p.Ser560* |  |  |  |  |
| 318 |  |  | c.1677G>A, p.Trp559* |  |  |  |  |
| 319 |  |  | c.1652G>A, p.Trp551* |  |  |  |  |
| 320 |  |  | c.1645_1648del, p.Asp549fs |  |  |  |  |
| 321 |  |  | c.1623_1630del, p.His542fs |  |  |  |  |
| 322 |  |  | c.1621C>T, p.Gln541* |  |  |  |  |
| 323 |  |  | c.1616_1617del, p.Thr539fs |  |  |  |  |
| 324 |  |  | c.1583dup, p.Ser528fs |  |  |  |  |
| 325 |  |  | c.1558del, p.Ala520fs |  |  |  |  |
| 326 |  |  | c.1536dup, p.Ser513fs |  |  |  |  |
| 327 |  |  | c.1523del, p.Val508fs |  |  |  |  |
| 328 |  |  | c.1518_1536del, p.Leu507fs |  |  |  |  |
| 329 |  |  | c.1477_1478del, p.Arg493fs |  |  |  |  |
| 330 |  |  | c.1462_1463del, p.Ser488fs |  |  |  |  |
| 331 |  |  | c.1444G>T, p.Glu482* |  |  |  |  |
| 332 |  |  | c.1422dup, p.Lys475fs |  |  |  |  |
| 333 |  |  | c.1401del, p.Arg468fs |  |  |  |  |
| 334 |  |  | c.1389del, p.Gly464fs |  |  |  |  |
| 335 |  |  | c.1381_1382del, p.Glu461fs |  |  |  |  |
| 336 |  |  | c.1355dup, p.Asn452fs |  |  |  |  |
| 337 |  |  | c.1355del, p.Asn452fs |  |  |  |  |
| 338 |  |  | c.1280_1281del, p.Arg427fs |  |  |  |  |
| 339 |  |  | c.1201_1204del, p.Lys401fs |  |  |  |  |
| 340 |  |  | c.1146dup, p.Ile383fs |  |  |  |  |
| 341 |  |  | c.1124_1128del, p.Thr375fs |  |  |  |  |
| 342 |  |  | c.1119del, p.Glu374fs |  |  |  |  |
| 343 |  |  | c.1063C>T, p.Gln355* |  |  |  |  |
| 344 |  |  | c.1018dup, p.Thr340fs |  |  |  |  |
| 345 |  |  | c.1009C>T, p.Gln337* |  |  |  |  |
| 346 |  |  | c.998del, p.Asn333fs |  |  |  |  |
| 347 |  |  | c.980_993del, p.Leu327fs |  |  |  |  |
| 348 |  |  | c.977del, p.Gly326fs |  |  |  |  |
| 349 |  |  | c.963dup, p.Glu322fs |  |  |  |  |
| 350 |  |  | c.928_934del, p.Pro310fs |  |  |  |  |
| 351 |  |  | c.894_916del, p.Glu298fs |  |  |  |  |
| 352 |  |  | c.893-1G>C |  |  |  |  |
| 353 |  |  | c.893-2A>G |  |  |  |  |
| 354 |  |  | c.867C>A, p.Tyr289* |  |  |  |  |
| 355 |  |  | c.769G>T, p.Gly257* |  |  |  |  |
| 356 |  |  | c.744+1G>T |  |  |  |  |
| 357 |  |  | c.744G>C, p.Lys248Asn |  |  |  |  |
| 358 |  |  | c.674C>T, p.Ala225Val |  |  |  |  |
| 359 |  |  | c.641dup, p.Tyr214* |  |  |  |  |
| 360 |  |  | c.637del, p.Tyr213fs |  |  |  |  |
| 361 |  |  | c.626G>A, p.Cys209Tyr |  |  |  |  |
| 362 |  |  | c.618del, p.His206fs |  |  |  |  |
| 363 |  |  | c.602-1G>A |  |  |  |  |
| 364 |  |  | c.600_601dup, p.Gly201fs |  |  |  |  |
| 365 |  |  | c.587_591del, p.Val196fs |  |  |  |  |
| 366 |  |  | c.568G>T, p.Glu190* |  |  |  |  |
| 367 |  |  | c.496G>T, p.Glu166* |  |  |  |  |
| 368 |  |  | c.488_501del, p.Lys163fs |  |  |  |  |
| 369 |  |  | c.439C>T, p.Gln147* |  |  |  |  |
| 370 |  |  | c.424C>T, p.Gln142* |  |  |  |  |
| 371 |  |  | c.397+1G>A |  |  |  |  |
| 372 |  |  | c.373G>A, p.Ala125Thr |  |  |  |  |
| 373 |  |  | c.323del, p.Gly108fs |  |  |  |  |
| 374 |  |  | c.291del, p.Phe98fs |  |  |  |  |
| 375 |  |  | c.277_283dup, p.Gly95fs |  |  |  |  |
| 376 |  |  | c.276_292del, p.Lys93fs |  |  |  |  |
| 377 |  |  | c.249del, p.Arg84fs |  |  |  |  |
| 378 |  |  | c.226+1G>A |  |  |  |  |
| 379 |  |  | c.226G>A, p.Glu76Lys |  |  |  |  |
| 380 |  |  | c.160C>T, p.Arg54* |  |  |  |  |
| 381 |  |  | c.99dup, p.Val34fs |  |  |  |  |
